# Analysis of clinical features, genomic landscapes and survival outcomes in HER2-low breast cancer

**DOI:** 10.1186/s12967-023-04076-9

**Published:** 2023-06-01

**Authors:** Juan Jin, Bin Li, Jianing Cao, Ting Li, Jian Zhang, Jun Cao, Mingchuan Zhao, Leiping Wang, Biyun Wang, Zhonghua Tao, Xichun Hu

**Affiliations:** 1 Department of Breast and Urologic Medical Oncology, Fudan University Shanghai Cancer Center, Shanghai, China; 2 Department of Oncology, Shanghai Medical College, Fudan University, Shanghai, China

**Keywords:** HER2-low breast cancer, Antibody–drug conjugates, HER2 expression, HER2 dynamics, Metastatic breast cancer, Heterogeneity

## Abstract

**Background:**

Novel human epidermal growth factor receptor 2 (HER2)-directed antibody–drug conjugates prompt the identification of the HER2-low subtype. However, the biological significance of HER2-low expression in breast cancer is unclear.

**Methods:**

Clinical and genomic data of 579 metastatic breast cancer patients were reviewed from our next-generation sequencing (NGS) database and genomic analysis of early breast cancer patients from TCGA was also analyzed.

**Findings:**

First, the clinicopathological characteristics of HER2-low patients were profoundly influenced by HR status and no difference of prognosis was observed between HER2-low and HER2-zero patients when paired by HR status, but notably HER2-low patients showed similar metastatic patterns to HER2-positive patients in the HR-positive (HR+ ) subgroup, with more brain and initial lung metastases and more cases of de novo stage IV breast cancer than HER2-zero patients. Second, among patients with primary HER2-low or HER2-zero tumors, the discordance of HER2 status between primary and metastatic tumors was significant, with 48.4% of patients with HER2-zero primary tumors exhibiting HER2-low phenotype in metastatic tumors in the HR+ subgroup. Third, within HR+ and HR-negative subtypes, HER2-low and HER2-zero tumors showed no substantial differences in mutation alterations and copy number variations. Forth, germline *BRCA2* mutations were observed only in HER2-low patients in our NGS database, especially in HR+ HER2-low tumors. Finally, three molecular subtypes based on genomic alterations in HER2-low breast cancer were identified, which provided novel insights into heterogeneity in HER2-low breast cancer.

**Conclusions:**

After correcting for HR expression, only marginal differences in clinical and molecular phenotypes were determined between HER2-low and HER2-zero breast cancer. Therefore, HER2-low breast cancer is insufficient to be defined as a distinct molecular entity, but rather a heterogenous disease.

**Supplementary Information:**

The online version contains supplementary material available at 10.1186/s12967-023-04076-9.

## Introduction

Breast cancer is highly heterogeneous and has been classified into 3 major molecular subtypes: hormone receptor (HR)-positive/human epidermal growth factor receptor (HER2)-negative, HER2-positive, and triple-negative [1]. The advent of the first anti-HER2 targeted agent, trastuzumab, remarkably improved the clinical outcomes of patients with HER2- positive breast cancer and at the same time contributed to the identification of HER2-positive breast cancer as a distinct biologic subtype [1, 2]. Previously, only patients with HER2-positive tumors, defined as tumors with an immunohistochemistry (IHC) score of 3+ or an IHC score of 2+/positive in situ hybridization (ISH) results, benefited from anti-HER2 targeted agents [2]. Currently, novel HER2-directed antibody-drug conjugate (ADC) agents, such as trastuzumab deruxtecan, not only show antitumor activity in classical HER2-positive breast cancer but also in HER2-low tumors, defined as tumors with an IHC score of 1+/2+ and negative ISH results [3–5]. HER2-targeted ADCs are designed to target HER2-expressing cancer cells and release chemotherapy agents inside cancer cells; the released chemotherapy agents can also be taken up by neighboring cancer cells without HER2 expression, causing cell death [6]. Based on the very favorable results of recent clinical trials, the anti-HER2 ADC trastuzumab deruxtecan has been approved for the treatment of metastatic HER2-low breast cancer, which emphasizes the importance of defining HER2-low tumors and promotes the investigation of biologic roles of HER2-low expression in breast cancer [3, 5].

According to previous reports, HER2-low breast cancer has a prevalence of 45–55% in the whole population, and tumors are more frequently HR-positive (HR+) [7–9]. In the phase III DESTINY-Breast04 clinical trial, the objective response rate of the novel ADC trastuzumab deruxtecan was more than 50% in pretreated HER2-low metastatic patients regardless of HR status [5]. The large subset of HER2-low patients and the impressive response in these patients spark our interest to more fully understand HER2-low expression in breast cancer. Many studies have investigated the clinicopathologic and molecular characteristics of HER2-low breast cancer, but no clear consensuses regarding prognostic effects have been reached, and intensive studies on mutation patterns in patients with different HER2 expression levels are still limited [7, 10, 11]. In addition, most previous retrospective studies focused on individual traits of HER2-low breast cancer, which makes understanding HER2-low breast cancer similar to collecting pieces of findings and might lead to a discrepancy in clinicopathological features and clinical outcomes extracted from different studies [8–15]. Therefore, a population with a comprehensive documentation, including clinicopathological characteristics, prognostic outcomes and genomic profiles, is urgently needed.

Here, we retrospectively analyzed 579 metastatic breast cancer patients from our next-generation sequencing (NGS) database with comprehensive clinicopathological features, including HER2 statuses in both primary and metastatic tumors, metastasis sites and clinical outcomes. In addition, genomic alterations were analyzed through biological data from 445/579 patients with appropriate ctDNA results, and the molecular diversity in HER2-low breast cancer was investigated to unravel the heterogeneity of the HER2-low disease. Early breast cancer patients from TCGA database were also used to analyze mRNA and mutation profiles as a supplemental analysis.

## Methods

### Study population and data extraction

*FUSCC database* Our NGS database is built from our China-Breast-Umbrella study initiated in September 2017, in which Chinese advanced breast cancer patients are planned to be treated with targeted therapy based on their mutation profiles; during the screening stage, the advanced patients undergo NGS testing of ctDNAs in Fudan University Shanghai Cancer Center (FUSCC). The Ethics Committee of FUSCC granted approval for this study (Approval Number: 1705172-9). All patients provided written informed consent for the use of blood and tissue samples for research. Genomic DNA isolated from venous blood was sequenced by NGS at the Burning Rock Biotech laboratory (Guangzhou, China), accredited by the College of American Pathologists and certified by the Clinical Laboratory Improvement Amendments, according to optimized protocols as described in detail previously [16]. Plasma samples were used to extract cell-free DNA (cfDNA), and germline DNA were extracted from white blood cells (WBCs). A minimum of 30 ng of DNA was used to construct NGS library. Target capture was performed using the commercial panels consisting of cancer-related genes. Indexed samples were sequenced on a NovaSeq 6000 (Illumina, Inc., CA, USA). Sequence data were mapped to the reference human genome (hg19) using Burrows-Wheeler Aligner (version 0.7.10). Copy number variations (CNVs) was calculated based on the ratio between the depth of coverage in tumor samples and average coverage of an adequate number (n > 50) of samples without CNV as references per capture interval. The germline variants were detected from the patient’s own WBCs and the somatic variant analyses filtered out these variants. Only germline mutations with pathogenic/likely pathogenic classifications according to the American College of Medical Genetics and Genomics were analyzed in our study. Tumor Mutational Burden (TMB) per sample in our study was computed as a ratio between nonsynonymous variants detected and the total coding region size of the gene panel, was described previously [16].

From November 2017 to September 2021, our NGS database included 445 patients with data from 520 cancer-related gene panels and 134 patients with other cancer-related gene panels. All patients were involved in the clinicopathological and prognostic analyses, and only genomic data from the 445/579 patients who underwent the 520 cancer-related gene panel were extracted for mutational and CNV analyses to avoid biases. The genes included in the 520 panel are listed in Additional file 1: Table S1.

The clinicopathological parameters of all patients from our NGS database, including age, pathological type, estrogen receptor (ER) status, progesterone receptor (PR) status, HER2 IHC score, HER2 fluorescence in situ hybridization (FISH) result, Ki67 score, details of metastatic sites, time of recurrence or metastasis and survival, were gathered from electronic medical records. All pathology results were confirmed by the Department of Pathology of FUSCC. Only patients from our database with a known HER2 IHC score and definitive FISH results when the HER2 IHC score was 2 + were included in the analysis. Disease free survival (DFS) is defined as the time from surgery to locoregional recurrence or distant metastasis. Overall survival (OS) is defined as the time from diagnosis of distant metastasis to death or last follow-up. The last follow-up time was April 20, 2022 and the median follow-up time of our NGS database was 32.6 months.

*TCGA database* Clinicopathological data from TCGA were downloaded by Xena Functional Genomics Explorer (https://xenabrowser.net). Masked somatic mutation data (VarScan2 Variant Aggregation and Masking) were obtained by TCGAbiolinks from TCGA GDC Data Portal (https://portal.gdc.cancer.gov). TMB per TCGA sample were downloaded from The Cancer Immunome Atlas (https://tcia.at/). HER2-positive, HER2-low and HER2-zero status in TCGA database was defined according to a previous study [11]. A total of 848 early breast cancer samples with available HER2 status were extracted from TCGA database, and the clinicopathological parameters were analyzed, as shown in Additional file 2: Table S2.

### HR and HER2 classification

HER2-positive status was defined as HER2 IHC score 3 + or 2 + with ISH amplification, according to the American Society of Clinical Oncology (ASCO)/College of American Pathologists (CAP) definition [17]. HER2-low status was defined as an IHC score of 1 + or 2 + without ISH amplification. HER2-zero status was defined as a HER2 IHC score of 0. In TCGA database, HER2-zero tumors also included tumors without an available IHC score but with a negative IHC status and a negative ISH amplification, consistent with the previous study [11]. Based on the IHC data, tumors with ≥ 1% of cancer cells with nuclear staining of ER or PR were considered as ER- or PR-positive and the positive ER or PR status of tumors was considered a positive HR status.

In our NGS database, the HER2 status of the primary tumor was used to investigate the differences in DFS and clinicopathological features, including age at diagnosis, T stage, N stage and pathology grade. The HER2 statuses used to analyze metastatic patterns, mutation profiles and CNV profiles in our NGS database were defined according to the most recent pathological results from primary tumors or metastatic biopsies. When analyzing the discordance of HER2 status in primary and metastatic breast cancer, HER2 status was determined from primary tumors or metastatic sites, and HR status was defined according to the primary tumors.

### Statistical analysis

The comparison of categorical variables was evaluated using the Chi-square test and Fisher’s exact test. The mean ± the standard error of the mean (SEM) and median ± 95% confidence intervals (CIs) were computed for continuous variables. Ordinary one-way ANOVA and Kruskal‒Wallis nonparametric tests were used to study the distribution of continuous variables across different groups. Survival curves were plotted using the Kaplan–Meier (KM) method and compared using the log-rank test. Univariate and multivariate Cox proportional hazard models were used to calculate hazard ratios and 95% CIs.

Clustering analysis was performed using nonnegative matrix factorization (NMF) based on Euclidean distance. The R package NMF was used to estimate the best rank using Lee’s algorithm with the following initial parameters: 2:8 for rank and numeric random seed (seed = 123,456). Analyses were performed using Graph Prism (version 9.4.1). All data were analyzed via the R statistics package (R version 4.1.2; R: The R-Project for Statistical Computing, Vienna, Austria). A P value of < 0.05 was considered significant.

## Results

### Patient cohorts and clinicopathological characteristics

**Fig. 1:**
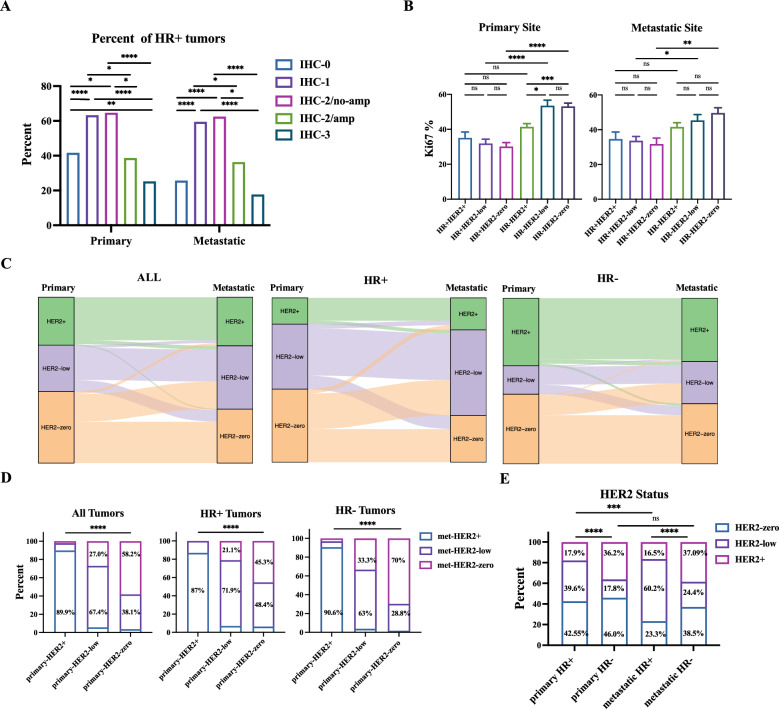
Clinicopathologic Characteristics according to HER2-low Status. **A** Frequency of HR+ disease according to HER2 phenotypes in primary and metastatic tumors. The *P* value was calculated by Chi-square test based on the number of cases per group featuring HR+ or HR− phenotype. Only the error bars with significant *P* values are shown. **B** Ki67 score stratified by HR and HER2 status in primary and metastatic tumors. The *P* value was calculated by T-test. **C** Distribution of HER2 status of primary and metastatic sites in the overall population, HR+ and HR− tumors. The Sankey diagram was mapped by the “ggalluvial” R package. **D** Absolute percentages of Fig. 1. C are reported. The *P* value was calculated by Chi-square test based on the number of cases per group featuring HER2-positive, HER2-low and HER2-zero phenotype. **E** The percentage of different HER2 statuses in HR+ and HR− subgroups of primary and metastatic breast cancer. The *P* value was calculated by Chi-square test based on the number of cases per group featuring HER2-positive, HER2-low and HER2-zero phenotype. amp: with HER2 gene amplification by FISH; no-amp: without HER2 gene amplification by FISH; met: metastatic; * 0.05 > p value ≥ 0.01, ** 0.01 > p value ≥ 0.001, *** p value < 0.001

A total of 579 metastatic breast cancer patients with available HER2 statuses from our NGS database were analyzed, including 495 patients with metastasis following radical operation and 84 patients with de novo stage IV breast cancer (Additional file 7: Fig. S1). HER2-low tumors were more often HR+, markedly higher than HER2-zero and HER2-positive tumors (63.19%, 42.02% and 28.00%, respectively, *p* < 0.0001) (Additional file 3: Table S3). The relationship between the percentage of HR + tumors and HER2 expression level seemed to be a parabolic distribution. A low percentage of HR + phenotype in HER2 IHC-0 and HER2 IHC-3 tumors, while a higher frequency of HR + phenotype in HER2 IHC-1 and IHC-2/no amplification tumors than tumors with other HER2 expression levels in both primary and metastatic tumors (Fig. 1A). Further study demonstrated that tumors with HER2 IHC-1 and IHC-2/no amplification contained higher percentages of ER- or PR-positive nuclear staining than other subgroups, although sometimes not pronounced (Additional file 8: Fig. S2A). Because of the high proportion of HR + tumors in HER2-low breast cancer, to avoid the influence of HR status on HER2-low subtype, we analyzed clinicopathological features divided by HER2 status of primary breast cancer within the HR + and HR-negative (HR−) subtypes (Table 1). Although HER2-low breast cancer patients had the lowest rate of lymph node metastasis, the subgroup analysis based on HR status showed no difference among different HER2 statuses in the HR+ or HR− subgroup, which suggested that the lower rate of lymphatic metastasis in HER2-low breast cancer was caused by the higher percentage of HR+ tumors (Table 1 and Additional file 3: Table S3). HR+ tumors had lower Ki67 scores than HR− tumors in both primary tumors and metastatic biopsies. In the HR− primary tumors, HER2-positive tumors had lower Ki67 scores than HER2-low and HER2-zero tumors, which indicated triple-negative breast cancer (TNBC) (Fig. 1B and Table 1). As 84 de novo stage IV patients were included in our study, we also analyzed the relationship between HER2 status and the stage at diagnosis. HER2-positive breast cancer was more likely to be de novo stage IV, and the proportion of de novo stage IV in HER2-low breast cancer was similar to HER2-positive breast cancer and more than that in HER2-zero breast cancer in all population or HR + subgroup (Table 1, Additional file 3: Table S3 and Additional file 8: Fig. S2B).

**Table 1:** Clinicopathological characteristics of patients stratified by HR and HER2 status on primary breast cancer

	HR+HER2-zero		HR+HER2-low		HR+HER2+		*p* value	HR−HER2-zero		HR−HER2-low		HR−HER2+		*P* value
	N = 100		N = 93		N = 42			N = 137		N = 53		N = 108		
Age, years														
≤ 45	53	53.00%	53	56.99%	20	47.62%	P vs L: ns Z vs L: ns	74	54.02%	27	50.94%	44	40.74%	P vs L: ns Z vs L: ns
> 45	47	47.00%	40	43.01%	22	52.38%		63	45.96%	26	49.06%	64	59.26%	
T stage														
pT1	28	40.00%	22	40.00%	9	34.62%	P vs L: ns Z vs L: ns	29	27.88%	14	35.90%	19	27.14%	P vs L: ns Z vs L: ns
pT2	38	54.29%	31	56.36%	13	50.00%		65	62.50%	22	56.41%	44	62.86%	
pT3-4	4	5.71%	2	3.64%	4	15.38%		10	9.62%	3	7.69%	7	10.00%	
N stage														
pN0	26	30.59%	27	39.13%	7	23.33%	P vs L: ns Z vs L: ns	35	29.66%	17	37.78%	19	23.17%	P vs L: ns Z vs L: ns
pN +	59	69.41%	42	60.87%	23	76.67%		83	70.34%	28	62.22%	63	76.83%	
Ki 67 percent (%)														
≤ 40	68	79.07%	56	74.67%	30	73.17%	P vs L: ns Z vs L: ns	44	34.65%	17	36.17%	61	60.40%	P vs L: 0.006 Z vs L: ns
> 40	18	20.93%	19	25.33%	11	26.83%		83	65.35%	30	63.83%	40	39.60%	
Pathology grade														
I–II	48	62.34%	42	65.63%	13	46.43%	P vs L: ns Z vs L: ns	24	23.08%	14	32.56%	29	36.25%	P vs L: ns Z vs L: ns
III	29	37.66%	22	34.37%	15	53.57%		80	76.92%	29	67.44%	51	63.75%	
De novo stage IV breast cancer														
Early	93	93.00%	73	78.49%	30	71.43%	P vs L: ns **Z vs L: 0.004**	124	90.51%	46	86.79%	86	79.63%	P vs L: ns Z vs L: ns
Advanced	7	7.00%	20	21.51%	12	28.57%		13	9.49%	7	13.21%	22	20.37%	

Bold value indicates the siginificant P values

HER2−: HER2-negative; HER2+: HER2-positive; HR−: HR-negative; HR+: HR-positive; P: HER2-positive; L: HER2-low; Z: HER2-zero;

The *P* value was calculated by Chi-square test

### Propensity of metastasis sites according to HER2 status

The metastatic sites among the different HER2 statuses were analyzed based on our records of the initial metastatic sites and metastatic sites at the last record (Table 2). The results showed that bone metastasis was more common in HER2-low breast cancer in the overall population, whether as initial metastatic sites or with disease progression, but not in the HR+ or HR− subgroups. Whether in the HR+ or HR− subtype, HER2-positive breast cancer showed the lowest incidence of bone metastasis. Brain metastasis is a key prognostic biomarker of breast cancer. In our study, HER2-positive patients had the highest frequency of brain metastasis in the overall population, consistent with previous reports [1]. In the HR+ subgroup, HER2-low breast cancer showed a significantly higher rate of brain metastasis than HER2-zero breast cancer (14.66% vs. 4.55%, *p* = 0.036). In addition, the frequency of initial metastasis to lung in HER2-low tumors was similar to that in HER2-positive tumors and was more frequent than that in HER2-zero tumors in HR+ subtype (25.76% vs. 42.24%, *p* = 0.026). With regard to liver metastasis, no difference was observed among different HER2 statuses in the subtype analysis by HR status.

**Table 2:** Metastatic Sites Stratified by HER2 Status

	HER2-zero N = 220		HER2-low N = 194		HER2+ N = 165		*P* value
Initial metastatic sites							
Lung	87	39.6%	79	40.7%	78	47.3%	P vs L: 0.22 Z vs L: 0.81
Liver	49	22.3%	52	26.8%	42	25.5%	P vs L: 0.77 Z vs L: 0.28
Bone	65	29.6%	81	41.8%	45	27.3%	P vs L: **0.004** Z vs L: **0.01**
Metastatic sites							
Brain	23	10.5%	29	15.0%	33	20.0%	P vs L: 0.21 Z vs L: 0.17
Lung	117	53.2%	98	50.6%	95	57.6%	P vs L: 0.18 Z vs L: 0.59
Liver	94	42.7%	105	54.1%	69	41.8%	P vs L: **0.02** Z vs L: **0.02**
Bone	113	51.4%	128	66.0%	66	40.0%	P vs L: < **0.0001** Z vs L:**0.003**

	HR+HER2-zero N = 66		HR+HER2-low N = 116		HR+HER2+ N = 42		*P* value
Initial metastatic sites							
Lung	17	**25.8%**	49	**42.2%**	20	47.6%	P vs L: 0.547 Z vs L:**0.026**
Liver	15	22.7%	30	25.9%	13	31.0%	P vs L: 0.525 Z vs L:0.637
Bone	32	48.5%	58	50.0%	20	47.6%	P vs L: 0.791 Z vs L: 0.844
Metastatic sites							
Brain	3	**4.6%**	17	**14.7%**	7	16.7%	P vs L: 0.756 Z vs L: 0.036
Lung	31	47.0%	63	54.3%	24	57.2%	P vs L: 0.75 Z vs L: 0.341
Liver	41	62.1%	70	60.3%	23	54.8%	P vs L: 0.529 Z vs L: 0.813
Bone	51	77.3%	90	**77.6%**	25	**59.5%**	P vs L: **0.024** Z vs L: 0.961

	HR–HER2-zero N = 154		HR–HER2-low N = 78		HR–HER2+ N = 123		*P* value
Initial metastatic sites							
Lung	70	45.45%	30	38.46%	58	47.15%	P vs L: 0.226 Z vs L: 0.31
Liver	34	22.08%	22	28.21%	29	23.58%	P vs L: 0.463 Z vs L: 0.303
Bone	33	21.43%	23	29.49%	25	20.33%	P vs L: 0.138 Z vs L: 0.175
Metastatic sites							
Brain	20	12.98%	12	15.38%	26	21.14%	P vs L: 0.31 Z vs L: 0.617
Lung	86	55.84%	35	44.87%	71	57.77%	P vs L: 0.075 Z vs L: 0.114
Liver	53	34.42%	35	44.87%	46	37.40%	P vs L: 0.292 Z vs L:0.121
Bone	62	40.26%	38	**48.72%**	41	**33.33%**	P vs L: **0.03** Z vs L:0.219

Bold value indicates the siginificant P values

HER2+: HER2-positive; HR−: HR-negative; HR+: HR-positive; P: HER2-positive; L: HER2-low; Z: HER2-zero;

HER2 Status was defined by the most recent pathological result

The *P* value was calculated by Chi-square test

### Distribution of HER2 status in primary tumors and metastatic tumors

In 301 metastatic breast cancer patients with the pathological results of both primary tumors and metastatic biopsies, we further determined the distribution of HER2 status in primary and metastatic tumors (Additional file 6: Fig. S1). In the whole cohort, the concordance rate of HER2-positive status between primary and metastatic tumors reached 89.9%, which showed the stability of HER2-positive status. Among patients with a primary HER2-low or HER2-zero tumor, the discordance of HER2 status between primary and metastatic tumors was much common. A total of 38.1% and 28.8% of patients with HER2-zero primary tumors exhibited HER2-low phenotype in metastatic tumors in the overall population and the HR− subtype, respectively, and the rate reached 48.4% in HR+ subtype. Conversely, 21.1% and 33.3% of patients with HER2-low primary tumors exhibited HER2-zero phenotype in metastatic tumors in the HR+ and HR− subtypes, respectively (Fig. 1C and D). This transformation pattern induced a higher proportion of HER2-low subtypes in metastatic tumors, especially in HR+ subtype (Fig. 1E).

### Survival analysis across HER2 subgroups

**Fig. 2:**
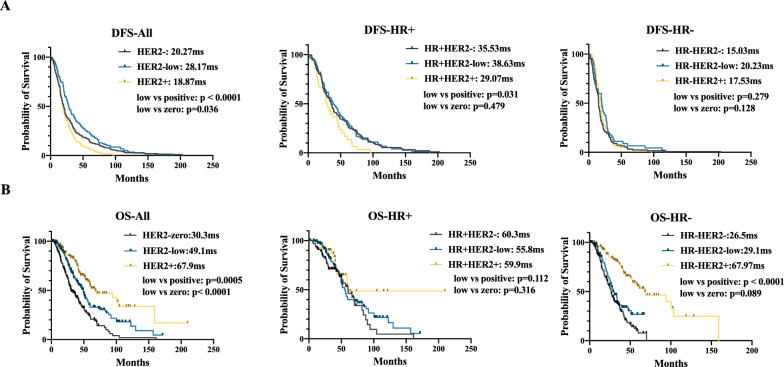
Kaplan–Meier Curves of DFS and OS. **A**, **B** DFS and OS were compared among different HER2 statuses in the overall population and the HR+ and HR− subgroups. Median DFS and OS are also represented. *P* value was calculated by Log-rank test

The KM analysis showed that the median DFS in HER2-positive, HER2-low and HER2-zero patients was 18.87, 28.17 and 20.27 months, respectively. Unexpectedly, HER2-low breast cancer patients had a longer DFS than both HER2-positive and HER2-zero groups (*P* < 0.0001 and *P* = 0.036, respectively). However, the subgroup analysis based on HR status did not reveal a significant difference in DFS between HER2-low and HER2-zero patients (Fig. 2A). Univariate Cox analysis also demonstrated favorable DFS in HER2-low tumors, and the trend was not pronounced in the HR+ and HR− subgroups. Larger tumor size, lymph node invasion, higher Ki67 score, HR− status and HER2-positive status predicted shorter DFS in all patients (Additional file 4: Table S4). In multivariable analyses including HR status, T stage, N stage, Ki67 score and HER2 status, no difference was observed between HER2-low and HER2-zero tumors (Table 3).

**Table 3:** Results from Multivariate Cox Proportional Hazard Models for DFS and OS

Parameter		HR (95% CI)	*P* value
DFS			
Ki67%		2.08 (1.53–2.82)	** < 0.001**
T stage	≤ T1 vs > T1	1.15 (0.90–1.47)	**0.269**
N stage	N− vs N+	1.38 (1.07–1.77)	**0.012**
HR status^a^	HR− vs HR+	0.51 (0.39–0.67)	** < 0.001**
HER2 status^a^	HER2-low vs HER2-zero	1.15 (0.86–1.54)	0.344
	HER2-low vs HER2+	1.00 (0.72–1.39)	0.997
OS			
Age		1.02 (1.01–1.03)	**0.003**
Ki67%		1.01 (1.002–1.014)	**0.007**
HR status^a^	HR− vs HR+	0.46 (0.35–0.62)	** < 0.001**
HER2 status^a^	HER2-low vs HER2-zero	1.40 (1.06–1.86)	**0.017**
	HER2-low vs HER2+	0.36 (0.26–0.51)	** < 0.001**
Initial metastasis sites	With visceral organ vs without	1.59 (1.13–2.23)	**0.007**
	With lung vs without	0.94 (0.69–1.28)	0.678

Bold value indicates the siginificant P values

DFS: disease free survival; OS: overall survival; HR status: hormone receptor status; HER2+: HER2-positive; HR: Hazard Ratio

^a^HR and HER2 status according to results on primary BC

Intriguingly, HER2-positive breast cancer was associated with longer OS than the other two subgroups in the overall population and the HR− subgroup. HER2-zero breast cancer patients had a shorter OS than HER2-low breast cancer patients (median OS: 30.3 vs. 49.1 months, *P* = 0.0005; HR: 1.72, 95% CI 1.33–2.22,* P* < 0.001) in the overall population by KM analysis and multivariate Cox analysis. However, in HR+ or HR− breast cancer, HER2-low patients and HER2-zero patients exhibited similar OS (Fig. 2B and Additional file 4: Table S4). Older age when diagnosed with recurrence or metastasis, HR− status, higher Ki67 score, and initial metastasis to visceral organ or lung was associated with shorter OS in the overall population (Additional file 4: Table S4). Further multivariable analyses, including age, HR status, Ki67 score and HER2 status and initial metastasis sites, showed that HER2-zero breast cancer patients had a worse OS than HER2-low breast cancer patients (HR: 1.48, 95% CI 1.12–1.95,* P* = 0.005) (Table 3).

### Mutation landscape among different HER2 statuses

**Fig. 3:**
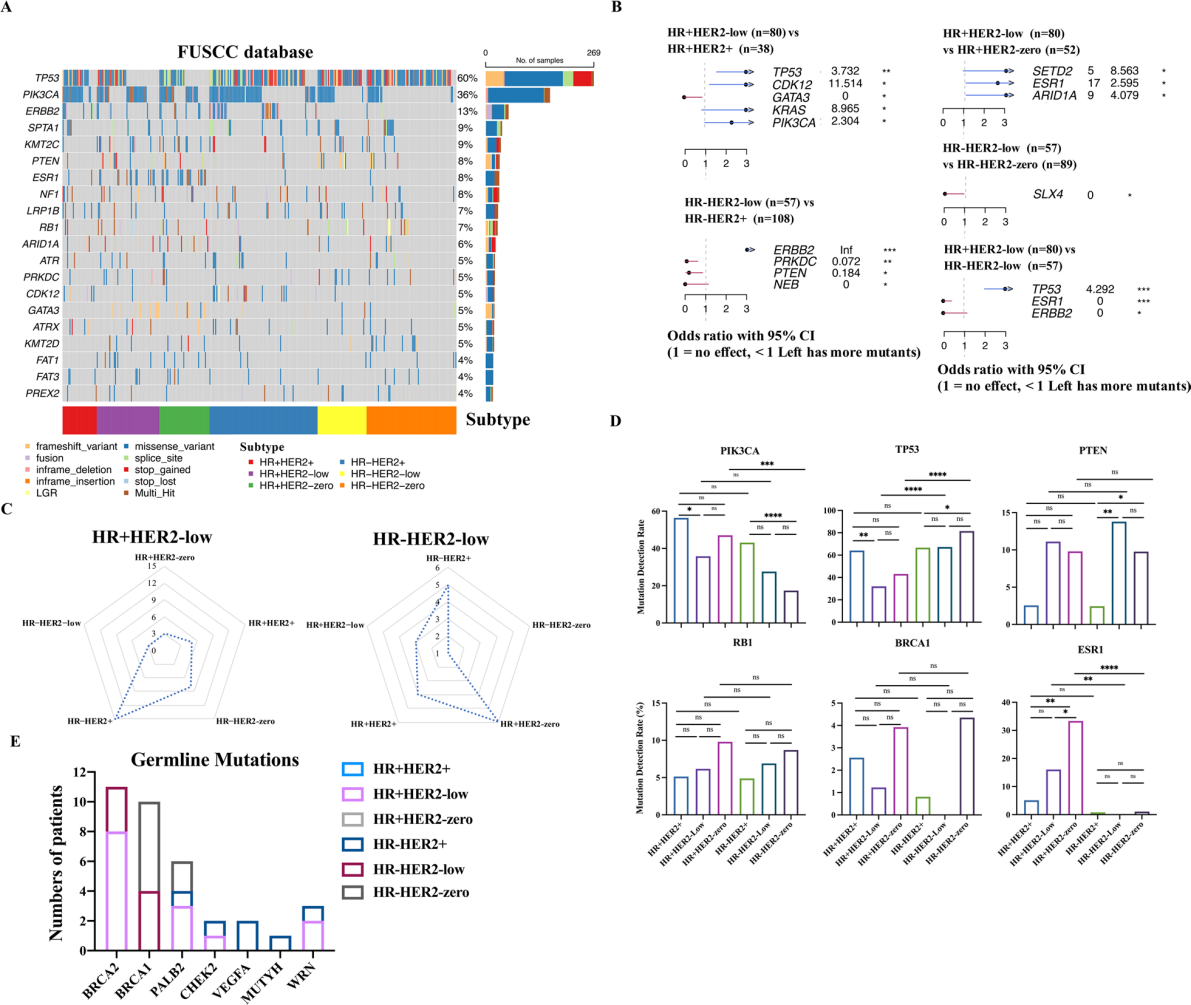
Mutation Profiles across Different HER2 Statuses. **A** Oncoplot of genomic alterations stratified by different subtypes from our NGS cohort. **B** The differentially mutated genes between different subtypes shown by forest plot in our NGS database. Only differentially mutated genes with statistical significance are shown, and the x-axis reports the odds ratio with 95% confidence intervals (CIs). < 1 represents more mutants in the left subtype, while > 1 represents more mutants in the right subtype. **C** Spider plot comparing the HR+HER2-low (left) or HR−HER2-low (right) group and the other subtypes. On the y-axis of the spider plot, the number of genes with a significantly different number of mutations (Fisher's exact test) between the reference group and each control group was reported. For instance, when considering the whole HR+HER2-low cohort, 5 differentially mutated genes with respect to the HR+HER2-positive cohort and 3 for the HR+HER2-zero cohort. When the whole HR−HER2-low cohort was compared with HR−HER2-positive tumors, we detected 5 differentially mutated genes with respect to the HR−HER2-positive cohort and 1 for the HR−HER2-zero cohort. **D**. The mutation rates of *PIK3CA*, *TP53*, *PTEN*, *RB1*, *BRCA1* and *ESR1* according to HR and HER2 status in our NGS database. The *P* value was calculated by the Chi-square test or Fisher's exact test based on the number of cases per group featuring the presence or absence of the gene mutation. **E** The number of patients with germline mutations in each subtype and in each individual gene. * 0.05 > p value ≥ 0.01, ** 0.01 > p value ≥ 0.001, *** 0.001 > p value ≥ 0.0001, **** p value < 0.0001

To further understand the molecular influence of HER2-low expression, NGS results of 445/579 patients from our database who underwent 520 cancer-related gene panel were analyzed. The prevalence of clinicopathological features among different HER2 statuses of these 445 patients was similar to the whole cohort, as shown in Additional file 5: Table S5. We also used the public database TCGA to help identify the mutation profiles as distributed among different subtypes. The waterfall plot showed the distribution of the mutation alterations according to HR and HER2 statuses in our database (Fig. 3A). Because clinical features of HER2-low breast cancer were profoundly influenced by HR status, therefore, the investigation of whether HER2-low tumors show a different mutation pattern compared to HER2-zero and HER2-positive tumors was also conducted within the HR+ and HR− subtypes. Considering the HER2-low breast cancer cohort, the differentially mutated genes compared to HER2-zero and HER2-positive patients in HR+ and HR− subtypes were recognized in our NGS database (Fig. 3B). When compared to HR+HER2-low breast cancer, HR+HER2-positive breast cancer had 5 differentially mutated genes (*TP53*, *CDK12*, *GATA3*, *KRAS* and *PIK3CA*) and HR+HER2-zero patients only had 3 ones (*SETD2*, *ESR1* and *ARID1A*). Likewise, HR−HER2-positive breast cancer showed more differentially mutated genes (*ERBB2*, *PRKDC*, *PTEN* and *NEB*) than HR−HER2-zero breast cancer (only *SLX4*) compared to HR−HER2-low breast cancer (3 vs 1) (Fig. 3B and C). Among HER2-low cohort, *TP53* mutations was enriched in HR−HER2-low patients, while *ESR1* and *ERBB2* mutations were more common in HR+HER2-low patients (Fig. 3B). Among the six subtypes defined by HR and HER2 statuses, it was proved again that HR+HER2-low breast cancer had less differently mutated genes with regard to HR+HER2-zero breast cancer than other subtypes and HR−HER2-low breast cancer was more like HR−HER2-zero breast cancer among these subtypes (Fig. 3C). The genes with the 15 highest mutation frequencies in HER2-positive, HER2-low and HER2-zero tumors were summarized, and the forest plots showed that the mutation rates of these genes in HER2-low and HER2-zero tumors were similar but different from those in HER2-positive patients, whether in our NGS database or TCGA database, in consistent with the above results (Additional file 9: Fig. S3A and B). The mutational frequencies of some crucial genes among different subtypes were represented in our NGS and TCGA databases (Fig. 3D and Additional file 9: Fig. S3C). *PIK3CA* mutations were more frequent in HR+ tumors, while *TP53* mutations occurred less prevalently in HR+ tumors. In HR− breast cancer, HER2-zero breast cancer harbored fewer *PIK3CA* mutations than HER2-positive and HER2-low breast cancer in the TCGA database. Intriguingly, subtype analysis showed that *TP53* mutations were more enriched in HR+HER2-positive than HR+HER2-low in both our NGS database and the TCGA database. *BRCA1* showed a higher mutation rate in HR− /HER2-zero breast cancer than that in HR−/HER2-low tumors, but without significant difference in both databases, which might suggest that HR−/HER2-zero breast cancer might be a more typical TNBC subtype than HR−/HER2-low tumors [18]. With regard to *ESR1* mutations, in HR+ breast cancer, HER2-zero tumors harbored the highest percentage of mutations than HER2-positive and HER2-low tumors in our NGS database. Although HER2-positive tumors harbored a higher TMB than HER2-low and HER2-zero tumors in HR+ breast cancer in the TCGA database, no difference in TMB among different subgroups was observed in our NGS database (Additional file 9: Fig. S3D).

The differences in germline mutations were also investigated, and the results showed that germline *BRCA2* mutations were found only in HER2-low subtype (11/139, 7.91%) and were more prevalent in HR+/HER2-low disease than HR−/HER2-low disease (8 vs 3 patients). *BRCA1* germline mutations were clustered in TNBC subtypes consisting of HR−/HER2-low and HR−/HER2-zero tumors, which was in accordance with a previous report (Fig. 3E) [19].

### CNV distribution and molecular clusters stratified by HER2 status

**Fig. 4:**
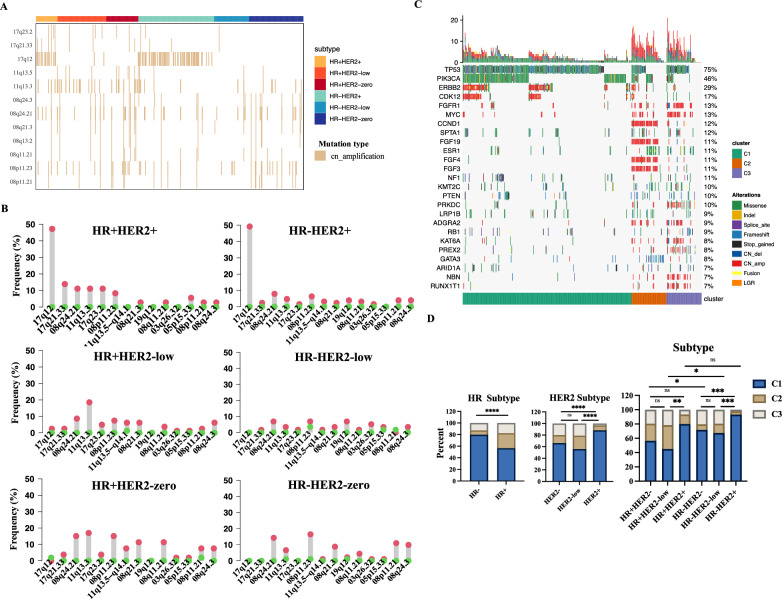
CNV profiles according to HER2 status. **A** The oncoplot of segments with an alteration frequency greater than 9% in the six subtypes in our NGS database. **B** The frequency distribution stratified by HR and HER2 status of the most common segments with amplifications from different subgroups in our NGS database. The lollipop plot was mapped by the “barplot” R package. **C** Oncoplot of the distribution of genomic alterations in different clusters in the whole cohort. **D** Comparison of the percentage of molecular clusters among the different subtypes. The *P* value was calculated by the Chi-square test based on the number of cases per group featuring the Cluster 1, Cluster 2 or Cluster 3 phenotype

As HER2 status is closely associated with *ERBB2* copy number variations (CNVs), CNV profiles are important for understanding the molecular properties of HER2 subgroups [20]. Segments with an alteration frequency greater than 9% in each subtype defined according to HR and HER2 status in our NGS database are shown in the oncoplot (Fig. 4A). The segments with the high amplification frequencies among the six subtypes are summarized (Fig. 4B). Whether in HR+ or HR− subtype, HER2-positive breast cancer was characterized by gains in 17q12 (*ERBB2, CDK12, HNF1B, RAD51D*), and almost no gains of 17q12 were present in the other subtypes. Frequency of gains in 17q12 was similar in HR+HER2-positive and HR−HER2-positive subtype (Fig. 4B). HER2-zero breast cancer was found to have gains in 08q24.21 (*MYC*) and 08p11.23 (*FGFR1*, *ADGRA2*, *ZNF703*). 11q13.3 (*CCND1*, *FGF19*, *FGF4*, *FGF3*) was significantly enriched in HR+ tumors independent of HER2 statuses. Analysis of oncogenes indicated that *ERBB2* (17q12) and *CDK12* (17q12) were especially amplified in the HER2-positive subgroup independent of HR status. No significant difference of gains in MYC (08q24.21) were found among the different subtypes in our NGS database. The gains of three cell cycle-related genes, *CCNE1* (19q12), *CCND1* (11q13.3) and *FGF4* (11q13.3), seemed be more associated with HR status than HER2 status (Additional file 10: Fig. S4A and B).

To further dissect the association between genomic signatures and molecular subtypes, we put together the mutation data and CNV data as a whole profile and conducted the NMF analysis based on the genomic profile of the whole population. Three distinct molecular clusters were identified (Additional file 10: Fig. S4C and Fig. 4C). Compared to HR+ breast cancer patients, HR− patients had more tumors with Cluster 1 phenotype. In whole population or HR+ or HR− subtype, HER2-positive patients had more tumors with Cluster 1 phenotype than HER2-low and HER2-zero patients and no difference in molecular clusters was observed between HER2-low and HER2-zero breast cancer patients (Fig. 4D). Conclusively, HER2-positive breast cancer had a significantly different molecular entity with HER2-low and HER2-zero breast cancer independent of HR status and HER2-low breast cancer and HER2-zero breast cancer shared a similar molecular signature even within the HR+ or HR− subtype.

### Molecular classification of HER2-low breast cancer

**Fig. 5:**
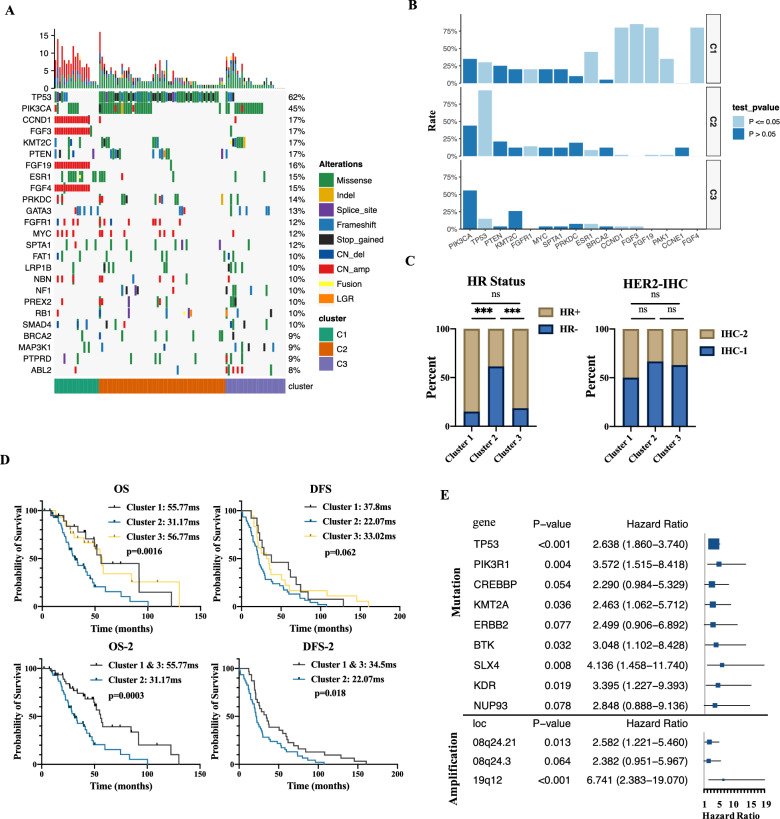
Identification of Molecular Subtypes in HER2-low Breast Cancer. **A** Oncoplot of distribution of genomic alterations in different clusters. **B** Mutation rates of genes stratified by the three clusters. The *P* value was calculated by Chi-square test or Fisher's exact test based on the number of cases per group featuring the presence or absence of the gene mutation. **C** Comparison of the percentage of HR+ tumors and HER2 IHC score among the three clusters. The *P* value was calculated by Chi-square test based on the number of cases per group featuring HR+ or HR− phenotype and the number of cases per group featuring IHC-1 or IHC-2 phenotype. **D** Kaplan‒Meier survival analysis of OS and DFS identified three subtypes. *P* value was calculated by Log-rank test. **E** Univariate Cox regression analysis for OS shown by forest plot (only those with p value < 0.1). The forest plot above the horizontal line showed the univariate Cox regression analysis of altered genes and the below showed the univariate Cox regression analysis of amplified segments

The above results suggested a complex clinical entity of HER2-low breast cancer. To further understand the molecular heterogeneity underlying HER2-low breast cancer, NMF analysis based on the genomic profile of the HER2-low subtype was performed. The most ideal value for cophenetic consensus was 3 (Additional file 11: Fig. S5A). The genomic landscape across the three molecularly distinct clusters is shown in Fig. 5A. Cluster 1 was enriched in *ESR1* mutations and *CCND1*, *FGF3*, *FGF19* and *FGF4* amplifications. Cluster 2 was enriched in *TP53* mutations. Cluster 3 was characterized by low *TP53* mutations and a lack of gene amplifications. No difference in the distribution of *PIK3CA* mutations existed among the three clusters (Fig. 5B).

Cluster 2 group included more HR− breast cancer patients than Cluster 1 and Cluster 3, and the distribution of HER2 IHC scores among different clusters showed no difference (Fig. 5C). In addition, the Ki67 scores, TMB, N stage, and number of initial metastatic sites were not different among the three clusters (Additional file 11: Fig. S5B). The survival analysis demonstrated that Cluster 2 had shorter OS and DFS than Cluster 1 and Cluster 3. When the Cluster 1 and Cluster 3 subgroups were combined into one group, the difference in OS and DFS between the combined group and Cluster 2 was more evident (median OS: 55.77 vs. 31.17 months, *p* = 0.0003; median DFS: 34.5 vs. 22.07 months,* p* = 0.018) (Fig. 5D). Multivariate Cox regression analyses, including age, Ki67 score, HR status and Cluster, demonstrated that both Cluster 2 and HR− status were associated with worse OS (Additional file 6: Table S6).

Univariate Cox regression analysis demonstrated that *TP53* mutations and 19q12 (*CCNE1*) amplifications predicted worse OS in breast cancer (Fig. 5E). In Cluster 2, *TP53* mutations and *CCNE1* amplifications exhibited a relatively higher frequency, which may be a reason for the worse clinical outcome of Cluster 2 (Fig. 5B).

## Discussion

Our study comprehensively investigated differences in clinicopathological parameters, mutation and CNV profiles among different HR and HER2 subgroups using our NGS database and TCGA database to exhaustively characterize the emerging HER2-low subtype.

The first conclusion drawn from the study was that HER2-low breast cancer was more likely to be HR+ , consistent with previous studies [7, 8, 11, 13, 15, 21]. It has already been demonstrated that HER2 expression is inversely related to ER and PR expression, but the correlation was not linear and was more complex [22]. Low expression of HER2 was significantly associated with HR+ expression compared to missing or positive HER2 expression. It was supposed that HER2 low expression promoted the expression of ER and PR, while high levels of HER2 suppressed the expression of ER and PR. The positive association between HR+ status and HER2-low status might provide a potential to combine HER2-targeted ADCs and endocrine therapies in HER2-low breast cancer. The high percentage of HR+ tumors induced the particular clinical behaviors in HER2-low breast cancer, including less postoperative nodal involvement and preference for bone metastasis. Intriguingly, HER2-positive and HER2-low breast cancers are more likely to be de novo stage IV breast cancers than HER2-zero tumors, especially in the HR+ subgroup. Higher frequencies of brain metastasis and initial metastasis into lung in HER2-low and HER2-positive breast cancer than HER2-zero breast cancer were observed in the HR+ subgroup. A recent study demonstrated a similar and increased risk of brain metastasis in HER2-low and HER2-positive breast cancer in the overall population and the HR+ subgroup [14]. In summary, the metastasis patterns of HER2-low tumors were more similar to those of HER2-positive tumors in HR+ breast cancer, although more retrospective studies for analysis and prospective studies for validation are needed.

An essential observation was that the significant discordance of HER2 status between primary and metastatic tumors among patients with primary HER2-low or HER2-zero tumors, highlighting the importance of reevaluating the HER2 status of relapse or metastatic biopsies. Previous studies demonstrated that the anti-tumor therapy including chemotherapy and radiotherapy could regulate HER2 expression and might promote novel HER2 status acquired during disease progression [23, 24]. But, intratumoral heterogeneity can also induce the discordance in HER2 expression between different regions or different samples from the same patient [25, 26]. It is difficult to figure out whether the discordance in HER2 expression between primary and metastatic tumors is an "evolution" acquired during disease progression or a manifestation of tumor heterogeneity. Therefore, the "evolution" may be an important mechanism underlying the discordance of HER2 status, and tumor heterogeneity is also a potential factor that cannot be ignored. In HR+ primary breast cancer, the translation rate of HER2-zero expression to HER2-low expression was much higher than the translation rate of HER2-low status to HER2-zero status, and therefore, more cases with HER2-low status emerged in HR+ breast cancer. Conversely, more cases of missing HER2-low status were observed in HR− breast cancer. The difference in the discordance of HER2 status between HR+ tumors and HR− tumors again highlights the crosstalk between HR+ expression and HER2-low status.

According to previous studies, the prognostic value of HER2-low status in breast cancer is contradictory. Schmidt’s retrospective study demonstrated a longer OS of patients with HER2-low tumors compared to those with HER2-zero tumors in the overall population and HR+ breast cancer patients [13, 27, 28]. Denkert’s recent study evaluating a cohort of breast cancer patients from four prospective clinical trials indicated that HER2-low disease was a favorable prognostic factor with superior DFS and OS compared to HER2-zero tumors only in the HR− subgroup [7]. Several other recent studies showed that there were no statistically significant differences in prognosis between HER2-low and HER2-zero breast cancer in HR+ or HR− subtype [8, 12, 15, 29, 30]. In our study, although HER2-low breast cancer patients had a longer DFS and OS than HER2-zero breast cancer patients in the overall population, subtype analysis based on HR status showed no difference. Given the favorable prognostic impact of positive HR expression in breast cancer, the better DFS and OS of HER2-low breast cancer might be attributed to the high proportion of HR+ tumors. Besides, all enrolled patients in our study developed metastatic disease, which suggested that these patients had aggressive prognostic factors, and not all patients received anti-HER2 adjuvant treatment due to expense, all of which might cause a shorter DFS in our study than previous results [31].

A recent retrospective study showed that in the GeparSepto trial, HER2-low breast cancer was associated with a higher frequency of *PIK3CA* mutations than HER2-zero tumors [7]. But our subtype analysis based on HR status did not show the difference. Both the GeparSepto study and our data showed that *TP53* mutations were more common in HER2-zero breast cancer than HER2-low breast cancer, but again the subtype analysis did not reveal any differences, which suggested that *TP53* mutations were notably influenced by HR expression rather than HER2 expression [7, 32]. In our analysis, after correcting for HR expression, HER2-positive breast cancer still showed significantly different mutations compared with HER2-low and HER2-zero breast cancer, but only marginal differences were found between HER2-low and HER2-zero tumors. Germline *BRCA2* mutations were found only in HER2-low patients in our NGS database, especially in patients with HR+ tumors, which might be a unique characteristic of HER2-low breast cancer. The observation was also in line with the previous report that *BRCA2* mutations are more common in ER-positive/ HER2-negative patients [18].

In our study, amplification of chromosome 17, in which *ERBB2* is located, was very common in HER2-positive tumors, which established the unique biological property of HER2-positive breast cancer and no particular CNVs enriched in HER2-low breast cancer were identified. HER2-low tumors harbored almost no *ERBB2* amplification and expressed HER2 protein in the tumor cell membrane by several mechanisms, including estrogen receptor pathways, the NF-kB pathway activated by chemoradiotherapy and epigenetic changes [10]. How to induce HER2 expression in tumor cells without *ERBB2* amplification will be a potential strategy for novel anti-HER2 treatments. Further analysis indicated that within the HR+ or HR− subtype, HER2-positive breast cancer revealed a different proportion of three molecular clusters divided by mutation and CNV profiles, while HER2-low and HER-zero breast cancer shared a similar proportion of three molecular clusters. The findings highlighted that HER2-low breast cancer are not substantially different from HER2-zero breast cancer in terms of genomic profiles.

The contradictory evidence of HER2-low breast cancer in clinical behaviors and molecular characteristics suggested the heterogeneity of HER2-low breast cancer. In our study, three distinct molecular subtypes in HER2-low breast cancer based on genomic profiles had their dominant characteristics and were associated with clinical survival. Cluster 2 enriched in *TP53* mutations was associated with a significantly worse prognosis. Although it highlights the molecular heterogeneity in HER2-low breast cancer, the diversity was still likely derived from the different HR expression, as *TP53* mutations corresponded to HR− tumors. The analysis based on HR status also demonstrated that Cluster 1 and Cluster 3 had similarly higher populations of HR+ breast cancer than Cluster 2. Whether classifying HER2-low breast cancer based on HR status is enough to recapitulate the molecular heterogeneity is unclear and needs further research. It is necessary to further classify HER2-low breast cancer for specializing treatment and developing appropriate molecular targeted therapies in different molecular subtypes.

The limitations of our study were obvious. (1) It was a retrospective study, with potential biases. (2) Low HER2 IHC scores were largely dependent on pathologists when low HER2 expression did not influence conventional anti-HER2 therapy without central pathological confirmation. (3) Timely pathological results when ctDNAs were collected were not available. Instead, the most recent pathological results were represented. As the most recent biopsies performed in most of the enrolled patients were after metastasis was diagnosed, the most recent pathological results were closest to the status when ctDNAs were collected. (4) The two databases used in our study were heterogeneous, making it impossible to compare or integrate them directly, and they were used only for mutual confirmation.

In conclusion, our study comprehensively investigated the clinical and genomic landscape of the HER2-low breast cancer within the HR+ and HR− subtypes in Chinese metastatic breast cancer patients. Positive feedback between low HER2 expression and positive HR expression might exist. The clinical behaviors and genetic alterations in HER2-low breast cancer were significantly influenced by HR status. Although the majority of clinical and molecular differences between HER2-low and HER2-zero breast cancer were no longer observed after correcting for HR status, some particular characteristics of HER2-low breast cancer compared to HER2-zero breast cancer still existed, including less *ESR1* mutations and more de novo stage IV cancers, brain metastasis and initial lung metastasis in the HR+ subgroup. The heterogeneity in HER2-low breast cancer was pronounced, and our elaboration of molecular subtypes in HER2-low breast cancer provided a further validation. Taken together, HER2-low breast cancer cannot be considered a distinct molecular entity and the subtypes HR+/HER2-low and HR−/HER2-low might be more suitable for clinical practice.

## Supplementary Information

**Additional file 1: Table S1.** The 520 Cancer-related Genes Included in the OncoScreen Plus Panel.

**Additional file 2: Table S2.** Clinicopathological Characteristics of Patients Stratified by HER2 Status in TCGA.

**Additional file 3: Table S3.** Clinicopathological Characteristics of Patients Stratified by HER2 Status in Primary Breast Cancer.

**Additional file 4: Table S4.** Results from Univariate Cox Proportional Hazard Models for DFS and OS.

**Additional file 5: Table S5.** Clinicopathological Characteristics of Patients with ctDNA Results Stratified by HER2 Status.

**Additional file 6: Table S6.** Results from Cox Proportional Hazard Models for OS in HER2-low Breast Cancer.

**Additional file 7: Figure S1.** Enrolled patients from FUSCC database in our analysis.

**Additional file 8: Figure S2.** Clinical Characteristics in Different HER2 Statuses. A. Percent of ER/PR-positive cells in tumors with different HER2 statuses in primary and metastatic tumors. The *P* value was calculated by T-test. Only the error bars with significant *P* values are shown. B. Comparison of de novo stage among the three HER2 subgroups. The *P* value was calculated by Chi-square test based on the number of cases per group featuring advanced and early phenotype.

**Additional file 9: Figure S3.** Mutation Profiles across Different HER2 Statuses in TCGA and FUSCC database. A. The difference in the high gene mutation frequencies among HER2-positive, HER2-low and HER2-zero subtypes shown by forest plot in the FUSCC database. The y-axis reports the genes with high mutation frequencies, and the x-axis reports the log odds ratio with 95% CIs. < 0 represents more mutants in the left subtype, while > 0 represents more mutants in the right subtype. B. The difference in the high gene mutation frequencies among HER2-positive, HER2-low and HER2-zero subtypes shown by forest plot in the TCGA database. The the y-axis reports the genes with high mutation frequencies, and x-axis reports the log odds ratio with 95% CIs. < 0 represents more mutants in the left subtype, while > 0 represents more mutants in the right subtype. C. The mutation rates of PIK3CA, TP53, PTEN, RB1, BRCA1 and ESR1 according to HR and HER2 status in the TCGA cohort. The P value was calculated by Chi-square test or Fisher's exact test based on the number of cases per group featuring the presence or absence of the gene mutation. D.TMB stratified by HR and HER2 status from our NGS and TCGA cohorts. The *P* value was calculated by nonparametric tests.

**Additional file 10: Figure S4.** Molecular Subtypes in the whole Breast Cancer Patients. A. Segment frequencies of *ERBB2*, *CDK12*, *MYC*, *CCNE1*, *CCND1* and *FGF4* distributed by HR and HER2 status in the FUSCC database. The *P* value was calculated by Chi-square test or Fisher's exact test based on the number of cases per group featuring the presence or absence of the gene amplification. B. Segment frequencies of *ERBB2*, *CDK12*, *MYC*, *CCNE1*, *CCND1* and *FGF4* distributed by HR and HER2 status in the TCGA database. The *P* value was calculated by Chi-square test or Fisher's exact test based on the number of cases per group featuring the presence or absence of the gene amplification. C. Associations between NMF coefficients and clustering numbers in the whole  breast cancer patients.

**Additional file 11: Figure S5.** Molecular Subtypes in HER2-low Breast Cancer. A. Associations between NMF coefficients and clustering numbers in HER2-low breast cancer. B. Comparison of the number of initial metastasis sites, N stage, Ki67 score and TMB among the three clusters.

## Data Availability

We encourage investigators interested in data sharing and collaboration to contact the corresponding author.

## Abbreviations

HER2: Human epidermal growth factor receptor 2
IHC: Immunohistochemistry
ISH: In­situ hybridization
HER2-positive: HER2-positive
HER2-zero: HER2-negative
ADC: Antibody–drug conjugate
ADCC: Antibody-dependent cell-mediated cytotoxicity
DFS: Disease free survival
CNV: Copy number variation
NGS: Next generation sequencing
CI: Confidence interval
FISH: Fluorescent in Situ Hybridization
HR–: Hormone receptor-negative
TMB: Tumor mutation load
CNG: Copy number gain
PCA: Principal components analysis
adj.P.Val: Adjusted P-value
HR +: Hormone receptor positive
OS: Overall survival
TNBC: Triple negative breast cancer
pCR: Pathologic complete response
