# Supplementary material for: Analysis of clinical features, genomic landscapes and survival outcomes in HER2-low breast cancer
Source: J Transl Med. 2023 Jun 1;21:360. doi: 10.1186/s12967-023-04076-9 (PMC10236705; doi:10.1186/s12967-023-04076-9)
Supplement: Supplementary file 1 — Additional file 1: Table S1. The 520 Cancer-related Genes Included in the OncoScreen Plus Panel. [file 12967_2023_4076_MOESM1_ESM.docx]

**Supplement Table 1. The 520 Cancer-related Genes Included in The OncoScreen Plus Panel**

| NTHL1 | BRCA1 | RPA1 | RUNX1 | TET2 | EIF4E | GATA4 | INHBA |
| --- | --- | --- | --- | --- | --- | --- | --- |
| PARP3 | NBN | ABL1 | RUNX1T1 | TGFBR1 | EMSY | GATA6 | INPP4A |
| MUTYH | RAD50 | ABL2 | SDC4 | TGFBR2 | EP300 | GID4 | INPP4B |
| PARP1 | RAD51 | ACVR1B | SDHA | TIPARP | EPCAM | GLI1 | INSR |
| PARP2 | RAD51B | AKT1 | SDHAF2 | TMEM127 | EPHA2 | GNA11 | IRF2 |
| ATM | RAD51D | AKT2 | SDHB | TMPRSS2 | EPHA3 | GNA13 | IRF4 |
| ATR | RAD52 | AKT3 | SDHC | TNFAIP3 | EPHA5 | GNAQ | IRS1 |
| CHEK1 | RAD54L | ALK | SDHD | TNFRSF14 | EPHA7 | GNAS | IRS2 |
| CHEK2 | XRCC2 | ALOX12B | SETD2 | TOP1 | EPHB1 | GPS2 | JAK1 |
| BRCA2 | XRCC3 | AMER1 | SF3B1 | TOP2A | ERBB2 | GREM1 | JAK2 |
| BRIP1 | GEN1 | APC | SGK1 | TP53 | ERBB3 | GRIN2A | JAK3 |
| FANCA | MRE11 | AR | SH2B3 | TP63 | ERBB4 | GRM3 | JUN |
| FANCC | MLH1 | ARAF | SH2D1A | TRAF2 | ERG | GSK3B | KAT6A |
| FANCD2 | MLH3 | ARFRP1 | SHQ1 | TRAF7 | ERRFI1 | H3F3A | KDM5A |
| FANCE | MSH2 | ARID1A | SLIT2 | TRIM58 | ESR1 | H3F3B | KDM5C |
| FANCF | MSH3 | ARID1B | SMAD2 | TSC1 | ETV6 | H3F3C | KDM6A |
| FANCG | MSH6 | ARID2 | SMAD3 | TSC2 | EWSR1 | HDAC1 | KDR |
| FANCI | PMS1 | ARID5B | SMAD4 | TSHR | EZH2 | HDAC2 | KEAP1 |
| FANCL | PMS2 | ASXL1 | SMARCA4 | U2AF1 | FAS | HGF | KEL |
| FANCM | PRKDC | ASXL2 | SMARCB1 | VEGFA | FAT1 | HIST1H1C | KIT |
| PALB2 | ERCC1 | ATRX | SMARCD1 | VEGFB | FBXW7 | HIST1H2BD | KLF4 |
| RAD51C | ERCC2 | AURKA | SMO | VHL | FGF10 | HIST1H3A | KLHL6 |
| SLX4 | ERCC3 | AURKB | SNCAIP | WISP3 | FGF12 | HIST1H3C | KMT2A |
| ABRAXAS1 | ERCC4 | AXIN1 | SOCS1 | WRN | FGF14 | HIST1H3D | KMT2C |
| BARD1 | ERCC5 | AXIN2 | SOX10 | WT1 | FGF19 | HIST1H3E | KMT2D |
| CCND3 | CRKL | AXL | SOX17 | XIAP | FGF23 | HIST1H3G | KRAS |
| CCNE1 | CRLF2 | B2M | SOX2 | XPO1 | FGF3 | HIST1H3H | LATS1 |
| CD274 | CSF1R | BAP1 | SOX9 | YAP1 | FGF4 | HIST1H3I | LATS2 |
| CD79A | CSF3R | BCL2 | SPEN | YES1 | FGF6 | HIST1H3J | LMO1 |
| CD79B | CSMD1 | BCL2L1 | SPOP | ZBTB16 | FGF7 | HIST2H3D | LRP1B |
| CDC73 | CSMD3 | BCL6 | SPTA1 | ZBTB2 | FGFR1 | HIST3H3 | LYN |
| CDH1 | CTCF | BCOR | SRC | ZNF217 | FGFR2 | HLA-A | MAGI2 |
| CDH18 | CTLA4 | BCORL1 | SRSF2 | ZNF703 | FGFR3 | HNF1A | MALT1 |
| CDK12 | CTNNA1 | BIRC3 | STAG2 | ZNRF3 | FGFR4 | HOXB13 | MAP2K1 |
| CDK4 | CTNNB1 | BLM | STAT3 | ACVR1 | FH | HRAS | MAP2K2 |
| CDK6 | CUL3 | BMPR1A | STAT4 | BBC3 | FLCN | HSD3B1 | MAP2K4 |
| CDK8 | CUL4A | BRAF | STAT5A | BCL10 | FLT1 | HSP90AA1 | MAP3K1 |
| CDKN1A | CXCR4 | BRD4 | STAT5B | BCL2L11 | FLT3 | ICOSLG | MAP3K13 |
| CDKN1B | CYLD | BRD7 | STK11 | BCL2L2 | FLT4 | ID3 | MAPK1 |
| CDKN1C | DAXX | BRINP3 | STK40 | CD74 | FOXA1 | IDH1 | MAPK3 |
| CDKN2A | DCUN1D1 | BTG1 | SUFU | CYP17A1 | FOXL2 | IDH2 | MAX |
| CDKN2B | DDR2 | BTG2 | SYK | CYP2D6 | FOXO1 | IFNGR1 | MCL1 |
| CDKN2C | DICER1 | BTK | TAF1 | DDR1 | FOXP1 | IGF1 | MDC1 |
| CEBPA | DNAJB1 | CALR | TBX3 | DIS3 | FRS2 | IGF1R | MDM2 |
| CENPA | DNMT3A | CARD11 | TCF3 | DNMT1 | FUBP1 | IGF2 | MDM4 |
| CHD1 | DNMT3B | CASP8 | TCF7L2 | DPYD | FYN | IKBKE | MED12 |
| CHD2 | DOT1L | CBFB | TENT5C | EPHB4 | GABRA6 | IKZF1 | MEF2B |
| CHD4 | EED | CBL | TERC | ETV4 | GATA1 | IL10 | MEN1 |
| CIC | EGFR | CCND1 | TERT | ETV5 | GATA2 | IL7R | MET |
| CREBBP | EIF1AX | CCND2 | TET1 | EZR | GATA3 | INHA | MGA |
| HLA-B | HLA-C | LTK | MAF | H3C2 | MERTK | MIR21 | NAV3 |
| NT5C2 | NUTM1 | PTPRO | RSPO2 | TRPC5 | TYRO3 | UGT1A1 | NFKBIA |
| PIK3C3 | PIK3CA | PIK3CB | PIK3CD | PIK3CG | PIK3R1 | PIK3R2 | PIK3R3 |
| PLCG2 | PLK2 | PNRC1 | POLD1 | POLE | PPARG | PPM1D | PPP2R1A |
| PPP6C | PRDM1 | PREX2 | PRKAR1A | PRKC1 | PRKN | PTCH1 | PTEN |
| PTPRS | PTPRT | QKI | RAB35 | RAC1 | RAD21 | RAF1 | RARA |
| RB1 | RBM10 | RECQL4 | REL | RET | RHEB | RHOA | RICTOR |
| RNF43 | ROS1 | RPS6KA4 | RPS6KB2 | RPTOR | RIT1 | PTPRD | RASA1 |
| MITF | MKNK1 | MPL | MST1 | MST1R | MTAP | MTOR | MYC |
| MYCL | MYCN | MYD88 | MYOD1 | NCOA3 | NCOR1 | NEGR1 | NF1 |
| NF2 | NFE2L2 | NFKB1A | NKX2-1 | NKX3-1 | NOTCH1 | NOTCH2 | NOTCH3 |
| NOTCH4 | NPM1 | NRAS | NRG1 | NSD1 | NSD2 | NTRK1 | NTRK2 |
| NTRK3 | NUP93 | P2RY8 | PAK1 | PAK3 | PAK5 | PAX5 | PBRM1 |
| PCDH11X | PDCD1 | PDCD1LG2 | PDGFRA | PDGFRB | PDK1 | PGR | PHOX2B |
| PIK3C2B | PIK3C2G | NCOR2 | NSD3 | PIM1 | PPP2R2A |  |  |
