# Supplementary material for: Analysis of clinical features, genomic landscapes and survival outcomes in HER2-low breast cancer
Source: J Transl Med. 2023 Jun 1;21:360. doi: 10.1186/s12967-023-04076-9 (PMC10236705; doi:10.1186/s12967-023-04076-9)
Supplement: Supplementary file 2 — Additional file 2: Table S2. Clinicopathological Characteristics of Patients Stratified by HER2 Status in TCGA. [file 12967_2023_4076_MOESM2_ESM.docx]

**Supplement Table 2. Clinicopathological Characteristics of Patients Stratified by HER2 Status in TCGA**

| subtype  total | HER2-zero  N=307 | | HER2-low  N=408 | | HER2+  N=133 | | *P* value |
| --- | --- | --- | --- | --- | --- | --- | --- |
| **Age, years** | | | | | | | |
| < 45 | 42 | 13.7% | 55 | 12.8% | 20 | 14.0% | 0.735 |
| 45 to <70 | 200 | 65.1% | 272 | 63.1% | 80 | 55.9% |  |
| ≥ 70 | 65 | 21.2% | 81 | 18.8% | 33 | 23.1% |  |
| **HR status** | | | | | | | |
| Negative | 70 | 23.0% | 73 | 17.9% | 32 | 24.2% | 0.138 |
| Positive | 234 | 77.0% | 335 | 82.1% | 100 | 75.8% |  |
| **T stage** | | | | | | | |
| pT1 | 84 | 27.5% | 109 | 26.7% | 26 | 19.5% | 0.404 |
| pT2 | 172 | 56.2% | 240 | 58.8% | 86 | 64.7% |  |
| pT3-4 | 50 | 16.3% | 59 | 14.5% | 21 | 15.8% |  |
| **N stage** | | | | | | | |
| pN0 | 161 | 53.0% | 207 | 51.5% | 48 | 36.9% | 0.005 |
| pN1 | 100 | 32.9% | 114 | 28.4% | 53 | 40.8% |  |
| pN2-3 | 43 | 14.1% | 81 | 20.1% | 29 | 22.3% |  |
| **Pathology** | | | | | | | |
| IDC | 111 | 83.5% | 296 | 72.5% | 205 | 67.0% | 0.01 |
| ILC | 18 | 13.5% | 78 | 19.1% | 61 | 19.9% |  |
| other | 3 | 2.3% | 27 | 6.6% | 33 | 10.8% |  |
| Mucinous Carcinoma | 1 | 0.8% | 7 | 1.7% | 7 | 2.3% |  |
| **PAM50** | | | | | | | |
| Basal-like | 61 | 25.6% | 51 | 15.4% | 2 | 1.9% | 0.0001 |
| HER2-enriched | 1 | 0.4% | 13 | 3.9% | 43 | 39.8% |  |
| Luminal A | 126 | 52.9% | 175 | 52.7% | 35 | 32.4% |  |
| Luminal B | 45 | 18.9% | 79 | 23.8% | 27 | 25.0% |  |
| Normal | 5 | 2.1% | 14 | 4.2% | 1 | 0.9% |  |

IDC: invasive ductal carcinoma; ILC: invasive lobular carcinoma;

HER2+: human epidermal growth factor receptor 2-positive; HR: hormone receptor;
