# Supplementary material for: Analysis of clinical features, genomic landscapes and survival outcomes in HER2-low breast cancer
Source: J Transl Med. 2023 Jun 1;21:360. doi: 10.1186/s12967-023-04076-9 (PMC10236705; doi:10.1186/s12967-023-04076-9)
Supplement: Supplementary file 3 — Additional file 3: Table S3. Clinicopathological Characteristics of Patients Stratified by HER2 Status in Primary Breast Cancer. [file 12967_2023_4076_MOESM3_ESM.docx]

**Supplement Table 3. Clinicopathological Characteristics of Patients Stratified by HER2 Status on Primary Breast Cancer**

|  | HER2-zero (N=237) | | HER2-low (N=146) | | HER2+ (N=150) | | *P* value |
| --- | --- | --- | --- | --- | --- | --- | --- |
| Age, years | | | | | | | |
| ≤ 45 | 127 | 53.58 % | 80 | 54.8% | 64 | 42.67% | P vs L: **0.048**  Z vs L: 0.818 |
| >45 | 110 | 46.42 % | 66 | 45.2% | 86 | 57.33% |  |
| HR status | | | | | | | |
| Negative | 137 | 57.8% | 53 | 36.3% | 108 | 72.00% | P vs L: **<0.0001**  Z vs L: **<0.0001** |
| Positive | 100 | 42.2% | 93 | 63.7% | 42 | 28.00% |  |
| T stage ^a^ | | | | | | | |
| pT1 | 57 | 33.14% | 37 | 38.95% | 28 | 29.17% | P vs L: 0.162  Z vs L: 0.55 |
| pT2 | 102 | 59.30% | 53 | 55.79% | 57 | 59.38% |  |
| pT3-4 | 13 | 7.56% | 5 | 5.26% | 11 | 11.46% |  |
| N stage ^b^ | | | | | | | |
| pN0 | 61 | 30.05% | 45 | 39.13% | 26 | 23.21% | P vs L: 0.099  Z vs L: **0.01** |
| pN+ | 142 | 69.95% | 70 | 60.87% | 86 | 76.79% |  |
| Ki67 percent (%) ^c^ | | | | | | | P vs L: 0.478  Z vs L:0.199 |
| ≤ 40 | 112 | 52.58% | 73 | 59.8% | 91 | 64.08% |  |
| > 40 | 101 | 47.42% | 49 | 40.2% | 51 | 35.92% |  |
| Grade ^d^ | | | | | | | |
| I-II | 67 | 38.0% | 56 | 52.34% | 42 | 38.89% | P vs L: **0.048**  Z vs L: **0.019** |
| III | 109 | 62.0% | 51 | 47.66% | 66 | 61.11% |  |
| Initial stage of breast cancer | | | | | | | |
| Early | 218 | 91.98% | 120 | 82.19% | 115 | 76.67% | P vs L: 0.24  Z vs L: **0.004** |
| Advanced | 19 | 8.02% | 26 | 17.81% | 35 | 23.33% |  |

a: 65, 50 and 55 samples of T stage in HER2－, HER2-low and HER2+ group were unevaluated respectively.

b: 35, 29 and 39 samples of N stage in HER2－, HER2-low and HER2+ group were unevaluated respectively.

c: 24, 24 and 8 samples of Ki67 in HER2－, HER2-low and HER2+ group were unevaluated respectively.

d: 56, 39 and 42 samples of pathology grade in HER2－, HER2-low and HER2+ group were unevaluated respectively.

HER2+: HER2-positive; pN+: with lymph node metastasis; P: positive; L: low; Z: zero;

The P value was calculated by Chi-square test.
