# Supplementary material for: Analysis of clinical features, genomic landscapes and survival outcomes in HER2-low breast cancer
Source: J Transl Med. 2023 Jun 1;21:360. doi: 10.1186/s12967-023-04076-9 (PMC10236705; doi:10.1186/s12967-023-04076-9)
Supplement: Supplementary file 4 — Additional file 4: Table S4. Results from Univariate Cox Proportional Hazard Models for DFS and OS. [file 12967_2023_4076_MOESM4_ESM.docx]

**Supplement Table 4. Results from Univariate COX Proportional Hazard Models for DFS and OS**

|  | **Parameter** | | **HR (95%CI)** | ***P* value** |
| --- | --- | --- | --- | --- |
| **DFS** | Age |  | 1.00 (0.99-1.01) | 0.972 |
|  | Ki67 % |  | 1.02 (1.01-1.02) | **<0.001** |
|  | T Stage | ≤ T1 vs >T1 | 1.40 (1.12-1.76) | **0.003** |
|  | N Stage | N- vs N+ | 1.36 (1.11-1.67) | **0.004** |
|  | HR Status^*^ | HR- vs HR+ | 0.42 (0.34-0.50) | **<0.001** |
|  | HER2 Status^*^ | HER2-low vs HER2-zero | 1.27 (1.01-1.59) | **0.038** |
|  |  | HER2-low vs HER2+ | 1.63 (1.26-2.12) | **<0.001** |
|  | HR+/HER2 Status | HR+HER2-low vs HR+HER2-zero | 1.02 (0.75-1.38) | 0.919 |
|  |  | HR+HER2-low vs HR+HER2+ | 1.56 (1.02-2.41) | **0.042** |
|  | HR-/HER2 Status | HR-HER2-low vs HR-HER2-zero | 1.32 (0.94-1.85) | 0.111 |
|  |  | HR-HER2-low vs HR-HER2+ | 1.14 (0.80-1.64) | 0.47 |
| **OS** | Age |  | 1.02 (1.01-1.03) | **0.005** |
|  | Ki67 % |  | 1.01 (1.01-1.02) | **<0.001** |
|  | HR Status^*^ | HR- vs HR+ | 0.52 (0.41-0.66) | **<0.001** |
|  | HER2 Status^*^ | HER2-low vs HER2-zero | 1.72 (1.33-2.22) | **<0.001** |
|  |  | HER2-low vs HER2+ | 0.59 (0.43-0.80) | **<0.001** |
|  | HR+/HER2 Status | HR+HER2-low vs HR+HER2-zero | 1.22 (0.81-1.86) | 0.343 |
|  |  | HR+HER2-low vs HR+HER2+ | 0.60 (0.32-1.12) | 0.109 |
|  | HR-/HER2 Status | HR-HER2-low vs HR-HER2-zero | 1.35 (0.95-1.91) | 0.092 |
|  |  | HR-HER2-low vs HR-HER2+ | 0.34 (0.22-0.52) | **<0.001** |
|  | Numbers of initial metastasis sites | ＜3 vs ≥3 | 1.30 (0.98-1.73) | 0.074 |
|  | Initial metastasis sites | with visceral organ vs without | 1.37 (1.08-1.75) | **0.011** |
|  |  | with bone vs without | 0.85 (0.67-1.07) | 0.165 |
|  |  | with liver vs without | 1.18 (0.92-1.51) | 0.198 |
|  |  | with lung vs without | 1.30 (1.04-1.62) | **0.021** |

* HR and HER2 status according to results on primary BC;

DFI: disease free interval; HR: hormone receptor; HR: Hazard Ratio;

HER2+: HER2-positive; HR－: HR-negative; HR2+: HR-positive;
