# Supplementary material for: Analysis of clinical features, genomic landscapes and survival outcomes in HER2-low breast cancer
Source: J Transl Med. 2023 Jun 1;21:360. doi: 10.1186/s12967-023-04076-9 (PMC10236705; doi:10.1186/s12967-023-04076-9)
Supplement: Supplementary file 5 — Additional file 5: Table S5. Clinicopathological Characteristics of Patients with ctDNA Results Stratified by HER2 Status. [file 12967_2023_4076_MOESM5_ESM.docx]

**Supplement Table 5. Clinicopathological Characteristics of Patients with ctDNA Results Stratified by HER2 Status**

|  | **HER2-zero (N=141)** | | **HER2-low**  **(N=137)** | | **HER2+**  **(N=146)** | | ***P* value** |
| --- | --- | --- | --- | --- | --- | --- | --- |
| **Age diagnosed breast cancer, years** | | | | | | | |
| ≤ 45 | 71 | 50.4% | 73 | 53.3% | 67 | 45.9% | positive vs low: 0.214  zero vs low: 0.625 |
| >45 | 70 | 49.6% | 64 | 46.7% | 79 | 54.1% |  |
| **HR status** | | | | | | | |
| Negative | 89 | 63.1% | 57 | 41.6% | 108 | 74.0% | positive vs low: **<0.0001**  zero vs low: **0.0003** |
| Positive | 52 | 36.9% | 80 | 58.4% | 38 | 26.0% |  |
| **Ki67 percent (%) ^a^** | | | | | | | positive vs low: 0.488  zero vs low: 0.541 |
| ≤ 40 | 76 | 56.3% | 78 | 60.0% | 91 | 64.1% |  |
| > 40 | 59 | 43.7% | 52 | 40.0% | 51 | 35.9% |  |
| **Initial stage of breast cancer** | | | | | | | |
| Early | 125 | 88.65% | 119 | 86.9% | 114 | 78.1% | positive vs low: 0.053  zero vs low: 0.649 |
| Advanced | 16 | 11.35% | 18 | 13.1% | 32 | 21.9% |  |

a: 8, 7 and 4 samples of Ki67 percent in HER2－, HER2-low and HER2+ group were unevaluated respectively.

HER2+: HER2-positive; pN+: with lymph node metastasis;

HER2 Status, HR status, Ki67 percent and pathology grade were defined by the most recent pathological results.

The P value was calculated by Chi-square test.
