# Supplementary material for: Analysis of clinical features, genomic landscapes and survival outcomes in HER2-low breast cancer
Source: J Transl Med. 2023 Jun 1;21:360. doi: 10.1186/s12967-023-04076-9 (PMC10236705; doi:10.1186/s12967-023-04076-9)
Supplement: Supplementary file 6 — Additional file 6: Table S6. Results from Cox Proportional Hazard Models for OS in HER2-low Breast Cancer. [file 12967_2023_4076_MOESM6_ESM.docx]

**Supplement Table 6. Results from COX Proportional Hazard Models for OS in HER2-low Breast Cancer**

|  | **Parameter** | | **HR (95%CI)** | ***P* value** |
| --- | --- | --- | --- | --- |
| **Univariate** | Age |  | 1.03 (1.01-1.05) | **0.001** |
|  | Ki67 % |  | 1.01 (1.01-1.02) | **0.017** |
|  | HR Status^*^ | HR- vs HR+ | 0.38 (0.25-0.58) | **<0.001** |
|  | Cluster | Cluster 2 vs Cluster 1&3 | 0.39 (0.22-0.66) | **<0.001** |
|  | Numbers of initial metastasis sites | ＜3 vs ≥3 | 1.25 (0.82-1.92) | 0.305 |
|  | Initial metastasis sites | with organ vs without | 1.30 (0.83-2.03) | 0.247 |
|  |  | with bone vs without | 0.94 (0.64-1.40) | 0.777 |
|  |  | with liver vs without | 1.27 (0.84-1.92) | 0.255 |
|  |  | with lung vs without | 1.12 (0.75-1.66) | 0.578 |
| **Multivariate** | Age |  | 1.02 (0.99-1.05) | 0.18 |
|  | Ki67 % |  | 1.007 (0.99-1.02) | 0.33 |
|  | HR Status^*^ | HR- vs HR+ | 0.46 (0.24-0.88) | **0.018** |
|  | Cluster | Cluster 2 vs Cluster 1&3 | 0.49 (0.25-095) | **0.034** |

* HR and HER2 status according to results on primary BC;

DFI: disease free interval; HR: hormone receptor; HR: Hazard Ratio;

HER2+: HER2-positive; HR－: HR-negative; HR2+: HR-positive;
