# Supplementary figures and images for: Analysis of clinical features, genomic landscapes and survival outcomes in HER2-low breast cancer
Source: J Transl Med. 2023 Jun 1;21:360. doi: 10.1186/s12967-023-04076-9 (PMC10236705; doi:10.1186/s12967-023-04076-9)

# Figure S1

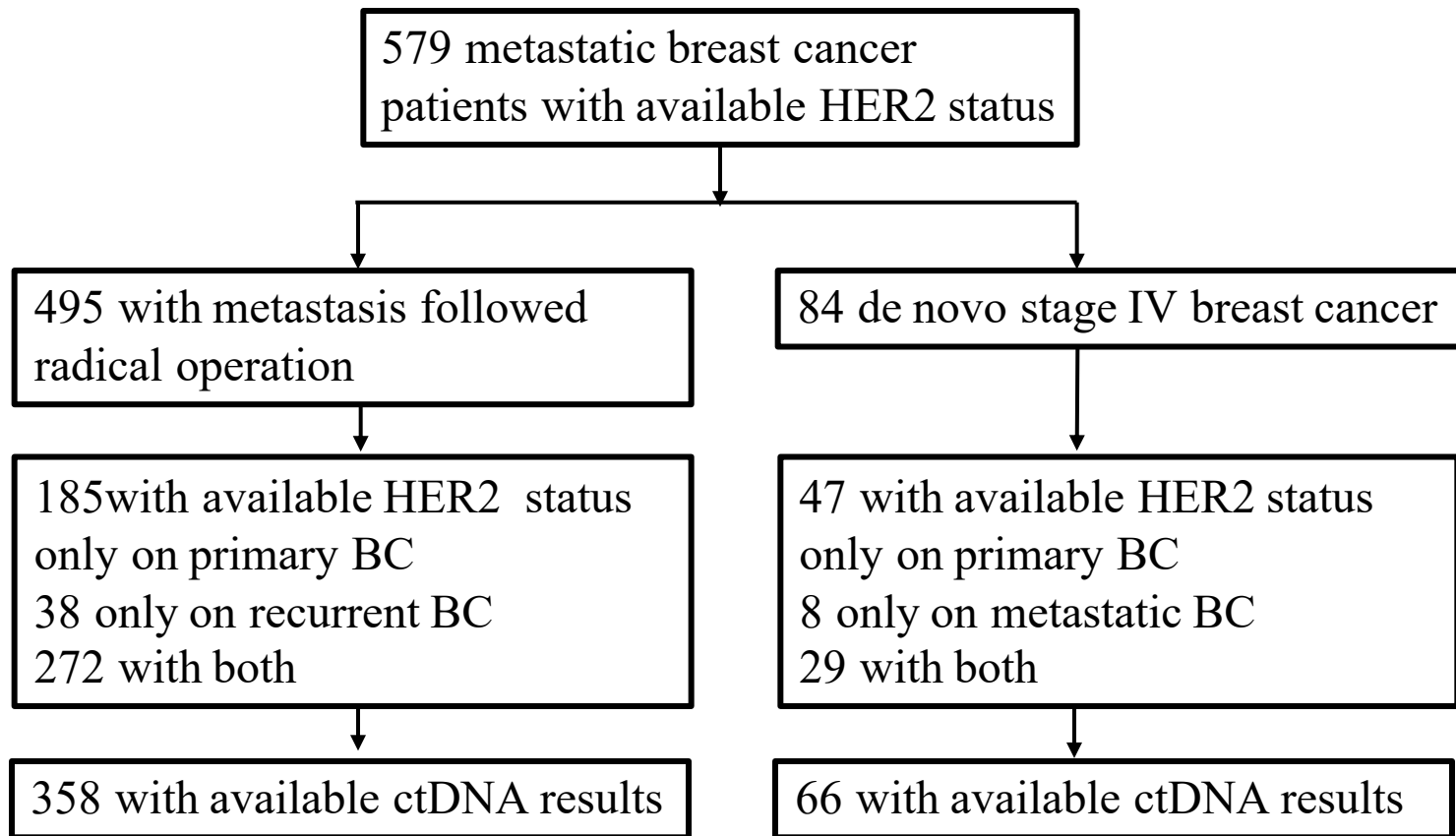

Supplement: Supplementary file 7 — Additional file 7: Figure S1. Enrolled patients from FUSCC database in our analysis. [file 12967_2023_4076_MOESM7_ESM.pdf]

Figure S2

A

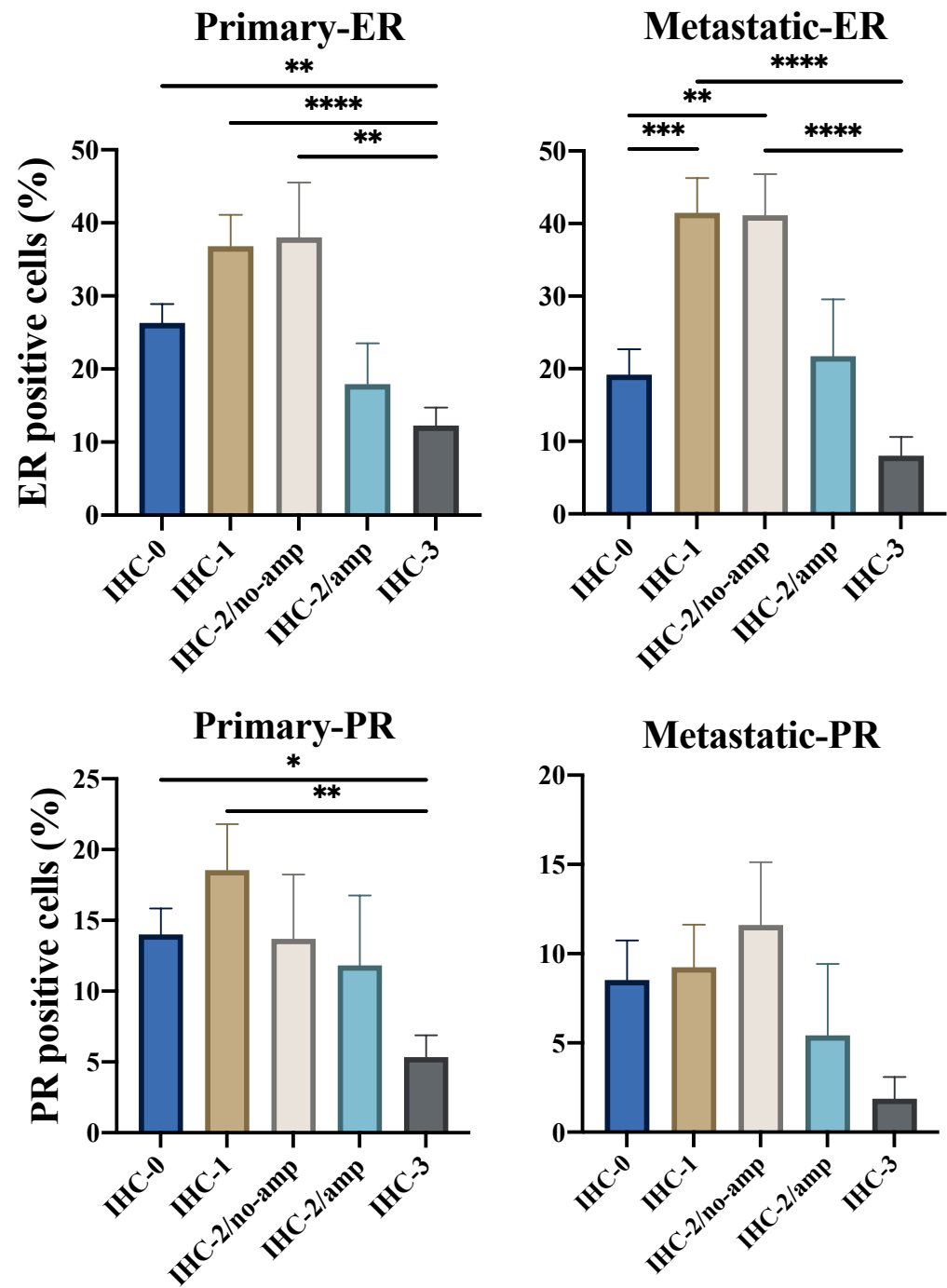

B

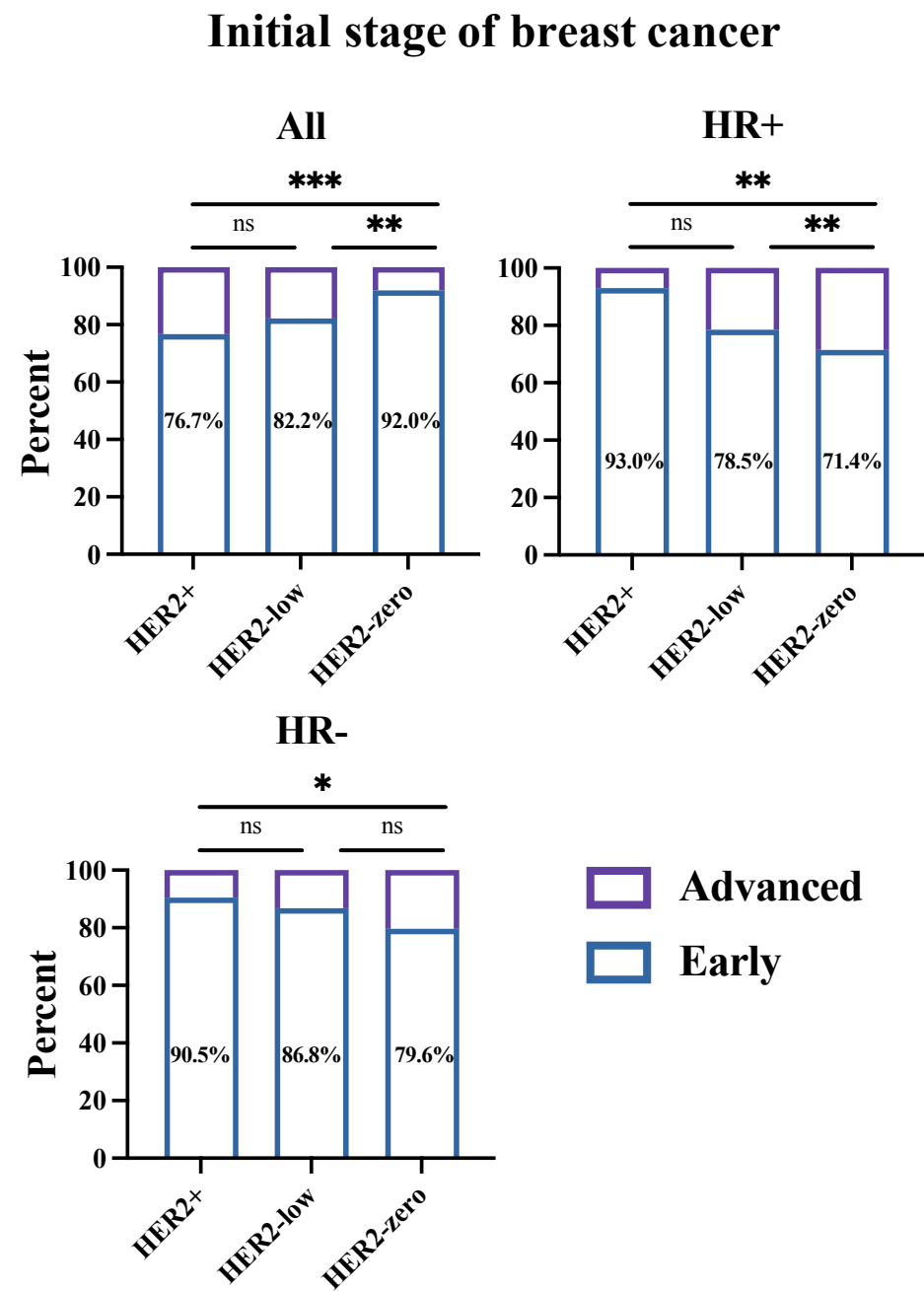

Supplement: Supplementary file 8 — Additional file 8: Figure S2. Clinical Characteristics in Different HER2 Statuses. A. Percent of ER/PR-positive cells in tumors with different HER2 statuses in primary and metastatic tumors. The P value was calculated by T-test. Only the error bars with significant P values are shown. B. Comparison of de novo stage among the three HER2 subgroups. The P value was calculated by Chi-square test based on the number of cases per group featuring advanced and early phenotype. [file 12967_2023_4076_MOESM8_ESM.pdf]

Figure S4

A

FUSCC database

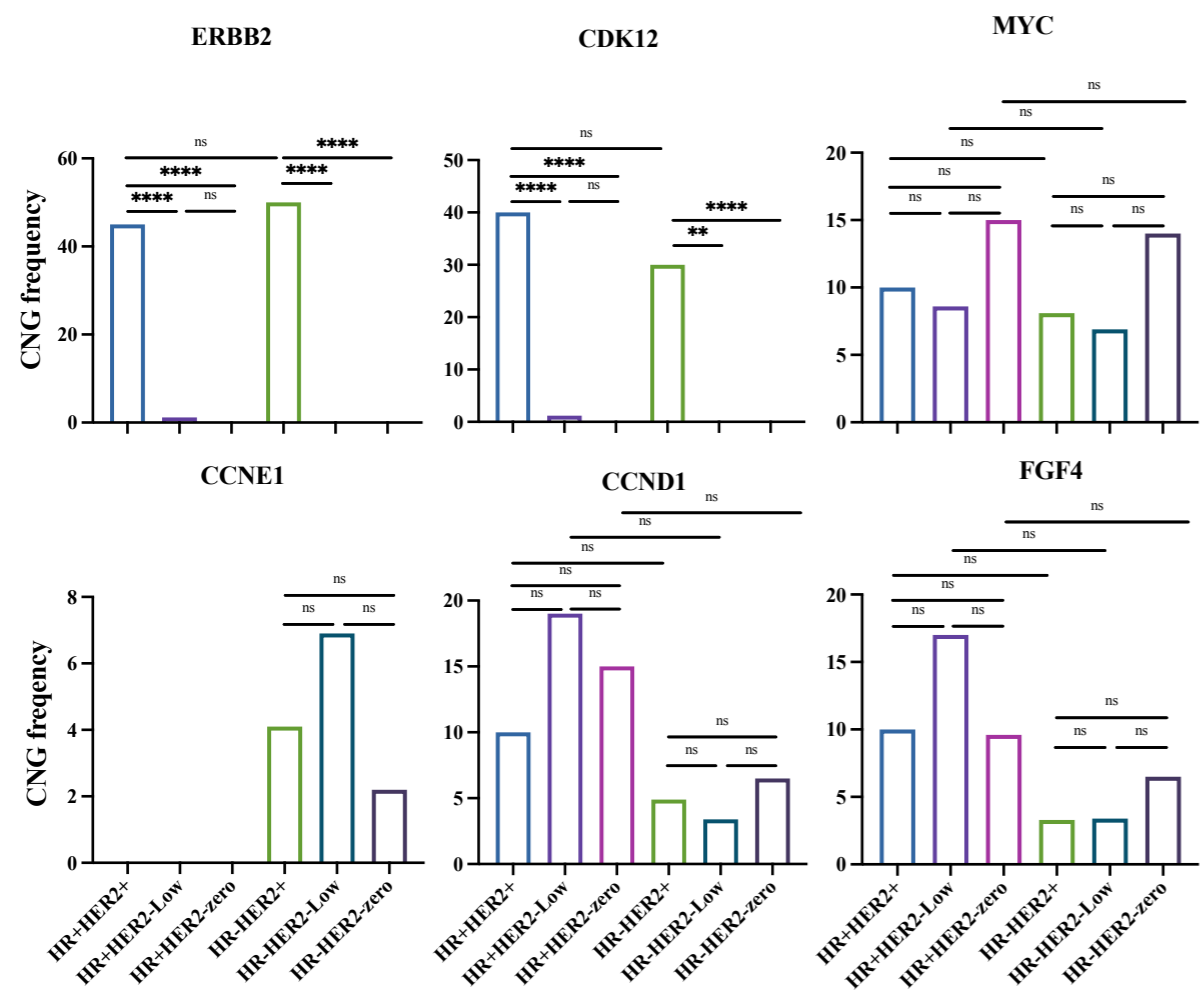

B

TCGA database

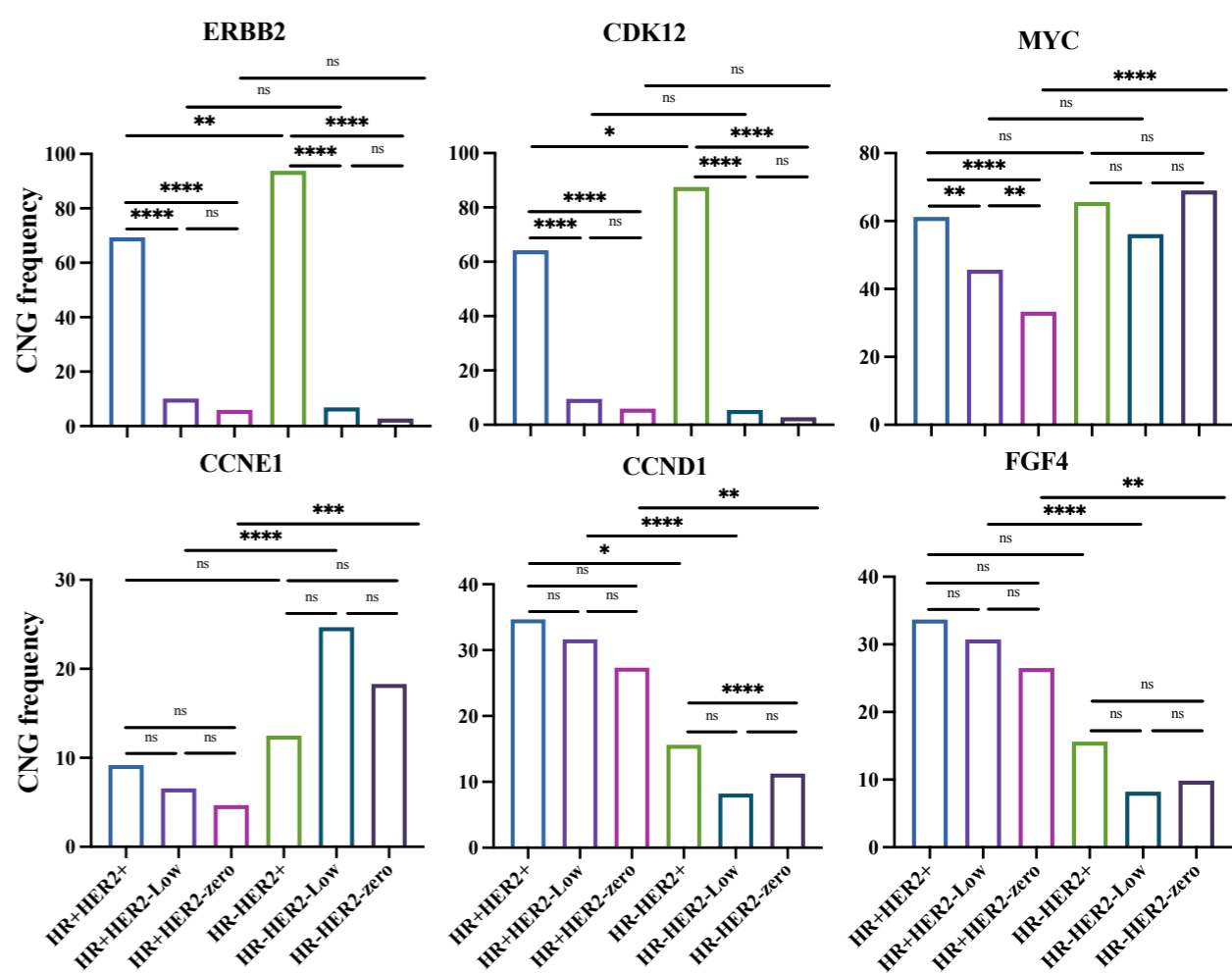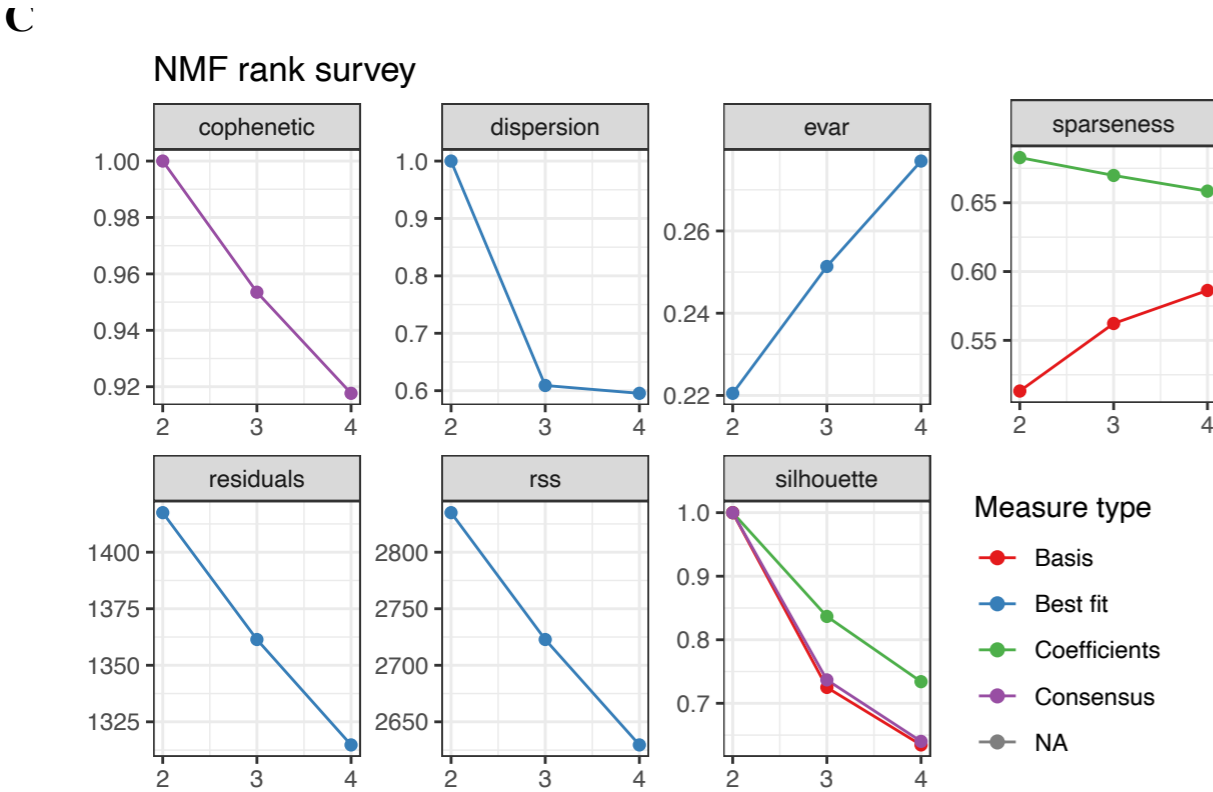

Supplement: Supplementary file 10 — Additional file 10: Figure S4. Molecular Subtypes in the whole Breast Cancer Patients. A. Segment frequencies of ERBB2, CDK12, MYC, CCNE1, CCND1 and FGF4 distributed by HR and HER2 status in the FUSCC database. The P value was calculated by Chi-square test or Fisher's exact test based on the number of cases per group featuring the presence or absence of the gene amplification. B. Segment frequencies of ERBB2, CDK12, MYC, CCNE1, CCND1 and FGF4 distributed by HR and HER2 status in the TCGA database. The P value was calculated by Chi-square test or Fisher's exact test based on the number of cases per group featuring the presence or absence of the gene amplification. C. Associations between NMF coefficients and clustering numbers in the whole breast cancer patients. [file 12967_2023_4076_MOESM10_ESM.pdf]

Figure S5

A

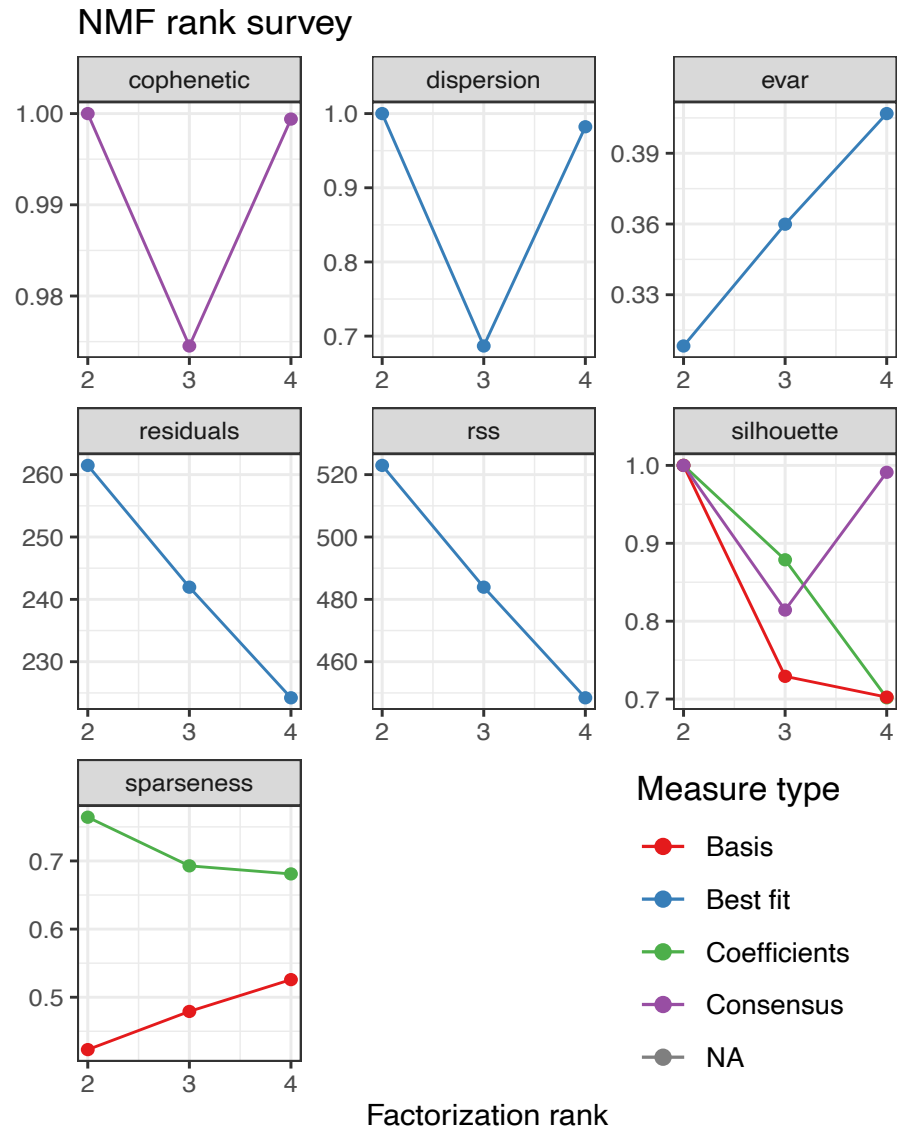

B

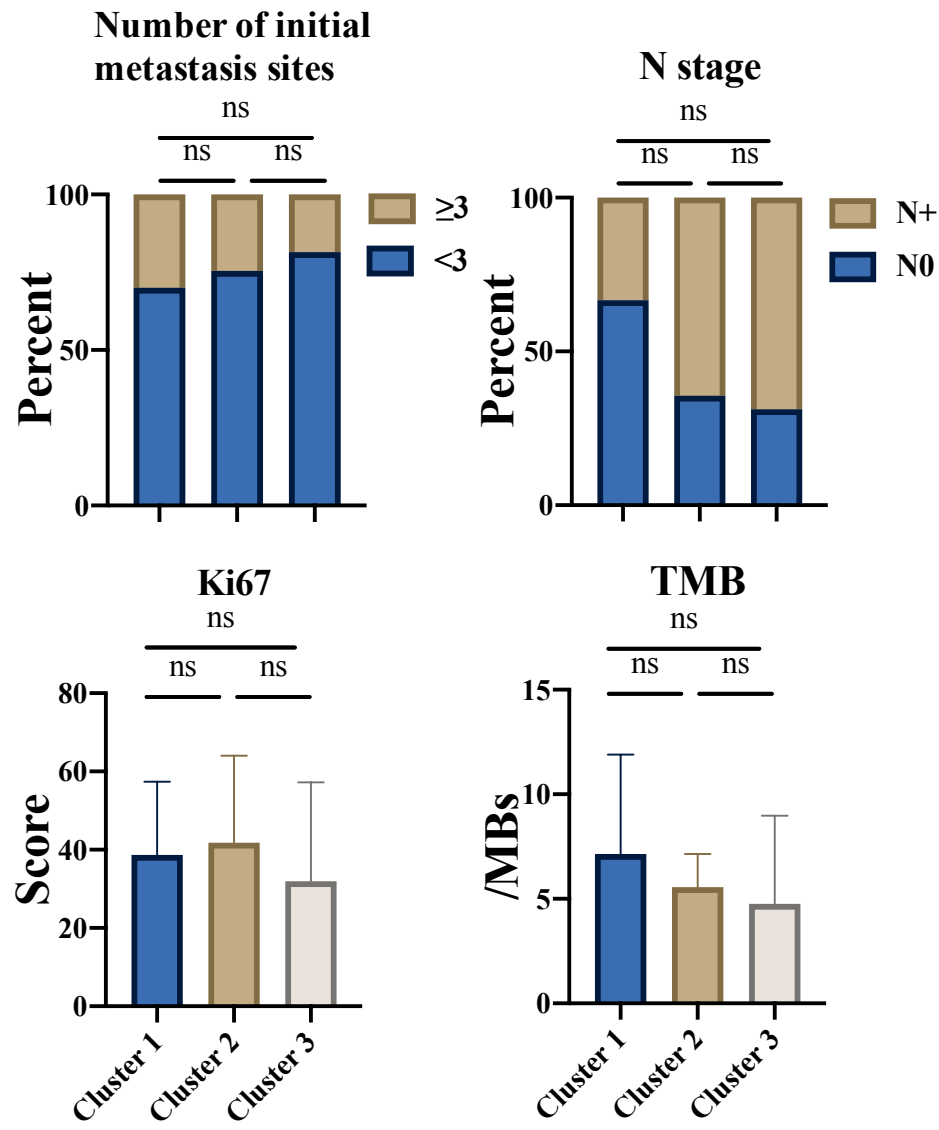

Supplement: Supplementary file 11 — Additional file 11: Figure S5. Molecular Subtypes in HER2-low Breast Cancer. A. Associations between NMF coefficients and clustering numbers in HER2-low breast cancer. B. Comparison of the number of initial metastasis sites, N stage, Ki67 score and TMB among the three clusters. [file 12967_2023_4076_MOESM11_ESM.pdf]
