# Supplementary material for: Analysis of clinical features, genomic landscapes and survival outcomes in HER2-low breast cancer
Source: J Transl Med. 2023 Jun 1;21:360. doi: 10.1186/s12967-023-04076-9 (PMC10236705; doi:10.1186/s12967-023-04076-9)
Supplement: Supplementary file 9 — Additional file 9: Figure S3. Mutation Profiles across Different HER2 Statuses in TCGA and FUSCC database. A. The difference in the high gene mutation frequencies among HER2-positive, HER2-low and HER2-zero subtypes shown by forest plot in the FUSCC database. The y-axis reports the genes with high mutation frequencies, and the x-axis reports the log odds ratio with 95% CIs. < 0 represents more mutants in the left subtype, while > 0 represents more mutants in the right subtype. B. The difference in the high gene mutation frequencies among HER2-positive, HER2-low and HER2-zero subtypes shown by forest plot in the TCGA database. The the y-axis reports the genes with high mutation frequencies, and x-axis reports the log odds ratio with 95% CIs. < 0 represents more mutants in the left subtype, while > 0 represents more mutants in the right subtype. C. The mutation rates of PIK3CA, TP53, PTEN, RB1, BRCA1 and ESR1 according to HR and HER2 status in the TCGA cohort. The P value was calculated by Chi-square test or Fisher's exact test based on the number of cases per group featuring the presence or absence of the gene mutation. D.TMB stratified by HR and HER2 status from our NGS and TCGA cohorts. The P value was calculated by nonparametric tests. [file 12967_2023_4076_MOESM9_ESM.pdf]

Figure S3

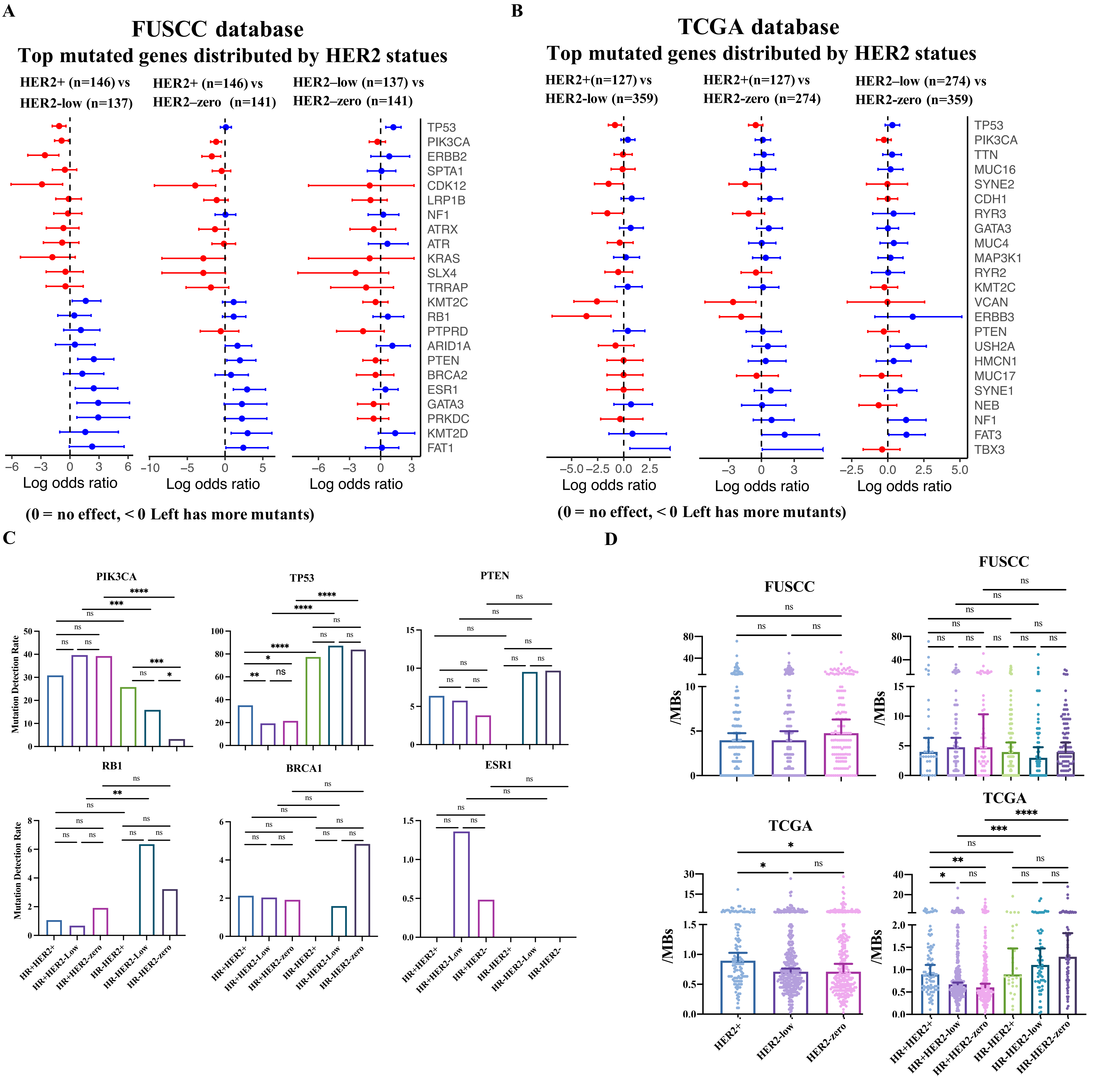
A
FUSCC database
Top mutated genes distributed by HER2 statues
HER2+ (n=146) vs HER2-low (n=137)
HER2+ (n=146) vs HER2–zero (n=141)
HER2–low (n=137) vs HER2–zero (n=141)
TP53
PIK3CA
ERBB2
SPTA1
CDK12
LRP1B
NF1
ATRX
ATR
KRAS
SLX4
TRRAP
KMT2C
RB1
PTPRD
ARID1A
PTEN
BRCA2
ESR1
GATA3
PRKDC
KMT2D
FAT1
Log odds ratio
(0 = no effect, < 0 Left has more mutants)
B
TCGA database
Top mutated genes distributed by HER2 statues
HER2+(n=127) vs HER2-low (n=359)
HER2+(n=127) vs HER2-zero (n=274)
HER2–low (n=274) vs HER2-zero (n=359)
TP53
PIK3CA
TTN
MUC16
SYNE2
CDH1
RYR3
GATA3
MUC4
MAP3K1
RYR2
KMT2C
VCAN
ERBB3
PTEN
USH2A
HMCN1
MUC17
SYNE1
NEB
NF1
FAT3
TBX3
Log odds ratio
(0 = no effect, < 0 Left has more mutants)
C
PIK3CA
TP53
PTEN
RB1
BRCA1
ESR1
Mutation Detection Rate
HR-HER2+
HR-HER2-Low
HR-HER2-zero
HR+HER2+
HR+HER2-Low
HR+HER2-zero
HR-HER2-
HR+HER2-
D
FUSCC
TCGA
/MBs
HER2+
HER2-low
HER2-zero
HR+HER2+
HR+HER2-low
HR+HER2-zero
HR-HER2+
HR-HER2-low
HR-HER2-zero
